# AI-driven routing and layered architectures for intelligent ICT in nanosensor networked systems

**DOI:** 10.1016/j.isci.2026.114626

**Published:** 2026-01-03

**Authors:** Alaa Kamal Yousif Dafhalla, Tahani Abdalla Attia Gasmalla, Ameni Filali, Nada Mohamed Osman Sid Ahmed, Tijjani Adam, Mohamed Elshaikh Elobaid, Subash Chandra Bose Gopinath

**Affiliations:** 1 Department of Computer Engineering, College of Computer Science and Engineering, University of Ha’il, Hail, Saudi Arabia; 2 Faculty of Electronic Engineering & Technology, Universiti Malaysia Perlis, Arau, Perlis 02600, Malaysia; 3 Micro System Technology, Centre of Excellence (CoE), Universiti Malaysia Perlis (UniMAP), Arau, Perlis, Malaysia; 4 Institute of Nano Electronic Engineering, Universiti Malaysia Perlis, Kangar, Perlis 01000, Malaysia; 5 Department of Neonatology, Saveetha Medical College and Hospital, Saveetha Institute of Medical and Technical Sciences, Chennai 602 105, Tamil Nadu, India; 6 Faculty of Chemical Engineering & Technology, Universiti Malaysia Perlis (UniMAP), Arau, Perlis 02600, Malaysia; 7 Department of Technical Sciences, Western Caspian University, Baku AZ 1075, Azerbaijan; 8 Department of Computer Science and Engineering, Faculty of Science and Information Technology, Daffodil International University, Daffodil Smart City, Birulia, Savar, Dhaka 1216, Bangladesh

**Keywords:** Applied sciences, Engineering, Sensor system

## Abstract

This review examines the emerging integration of nanosensor networks with modern information and communication technologies to address critical needs in healthcare, environmental monitoring, and smart infrastructure. It evaluates how machine learning and artificial intelligence techniques improve data processing, energy management, real-time communication, and scalable system coordination within nanosensor environments. The analysis compares major learning approaches, including supervised, unsupervised, reinforcement, and deep learning methods, and highlights their effectiveness in data routing, anomaly detection, security, and predictive maintenance. The review also assesses new system architectures based on edge computing, cloud federated models, and intelligent communication protocols, focusing on performance indicators such as latency, throughput, and energy efficiency. Key challenges involving computational load, data privacy, and system interoperability are identified, and potential solutions inspired by biological systems, interpretable models, and quantum-based learning are explored. Overall, this work provides a unified framework for advancing intelligent and resource-efficient nanosensor communication systems with broad societal impact.

## Graphical abstract

**Figure:**
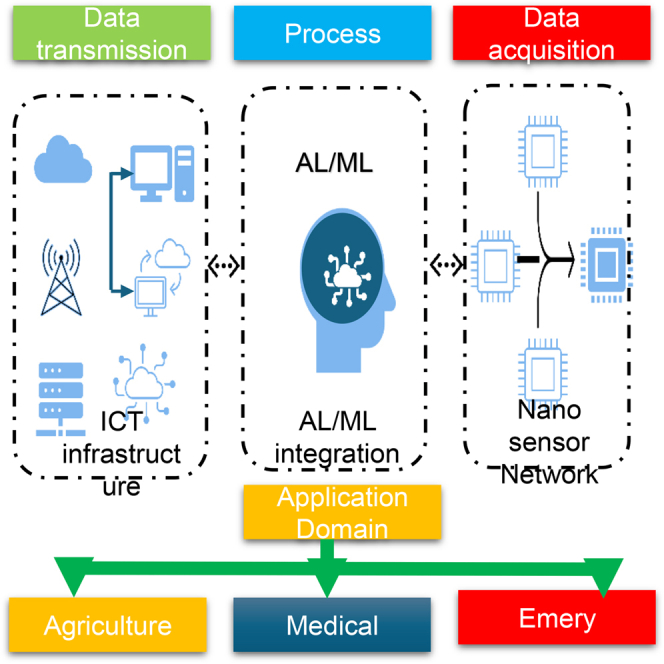

## Introduction

Rapid developments in nanosensors—miniaturized sensing devices capable of detecting molecular-level changes—have gained tremendous attention.^1^ The underlying atomic-scale phenomena enable precise engineering of their surfaces and architectures, establishing nanosensors as pivotal components in the evolution of modern sensing technologies and their seamless integration with information and communication technology (ICT) systems.^1^^,^^2^ These sensors exhibit unique characteristics such as high sensitivity, rapid response time, and adaptability for networked or embedded environments, making them highly suitable for real-time monitoring applications.^3^ The growing demand across diverse sectors—including biomedical diagnostics, environmental surveillance, agriculture, smart cities, and defense systems—has intensified efforts toward developing integrated ICT-enabled nanosensor systems capable of performing autonomously and with high accuracy.^4^ Within networked ICT infrastructures, nanosensors play a transformative role in enabling intelligent, interconnected, and data-driven operations. Nanosensors allow and facilitate seamless data collections, enabling intelligent systems to perceive, process, and react to dynamic conditions at micro and nano scales.^5^ To use the full potential of nanosensor networks, there is a growing need toward integrating Artificial Intelligence (AI) and Machine Learning (ML) into ICT frameworks.^6^ These approaches will allow powerful tools for handling the massive volume and complexity of data generated by nanosensors.^7^ Recent advances in edge computing and federated learning (FL) enable low-latency, privacy-preserving, and scalable distributed intelligence by training models directly on edge devices without sharing raw data. Moreover, deep reinforcement learning (DRL) have also made it feasible to embed intelligence closer to the sensor nodes. On the other hand, it enables decentralized decision making, adaptive system behavior, predictive maintenance, and anomaly detection.^8^ This trend is redefining traditional ICT paradigms, transforming passive nanosensor systems into proactive, self-optimizing networks.^9^ The approach has tremendous advancements; even with this progress, several critical challenges remain, which need serious attention. Nanosensor-enabled networks often operate under stringent protocols and constraints.^10^ Due to limited computational capacity, energy scarcity, bandwidth limitations, and real-time responsiveness they face unlimited constraints. Moreover, the heterogeneous nature of sensor data, the dynamic topology of sensor networks, and security vulnerabilities further complicate the integration of ML/AI into existing ICT architectures.^11^ Several challenges such as issues related to scalability, model generalization, data privacy, and hardware compatibility also hinder widespread adoption.^12^ This review aims to provide a comprehensive and systematic analysis of the current state of the art in ML and AI integration for nanosensor-based ICT systems. We explore various ML paradigms, AI-driven network architectures, and performance enhancement techniques.^13^ The review categorizes and compares existing methods, highlights application-specific use cases, and identifies key performance indicators.^14^ Furthermore, it investigates the limitations of current approaches and proposes future directions involving edge intelligence, explainable AI, and sustainable computing for nanosensor networks.^15^ This study introduces a novel AI-driven framework that integrates miniaturized nanosensor networks with optimized ICT systems to address key challenges in real-time data processing, energy efficiency, and communication reliability. Unlike existing models that focus narrowly on either sensor performance or network protocols, this framework combines hybrid ML techniques including supervised, unsupervised, and RL with edge computing strategies to enable adaptive, scalable, and low-latency decision-making in complex environments. The contribution is further strengthened by benchmarking the framework using multiple publicly available datasets across healthcare, environmental monitoring, and smart infrastructure domains, ensuring reproducibility and cross-domain applicability. This work not only advances the design of intelligent nanosensor networks but also lays the groundwork for next-generation cyber-physical systems with practical impact across critical sectors.

**Figure 1:**
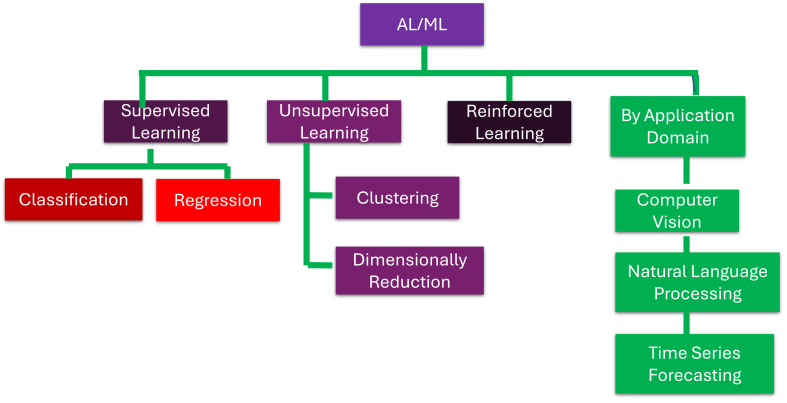
Taxonomy of AI/ML approaches based on learning types and key application domains

The taxonomy present in Figure 1 categorizes AI/ML methodologies into four principal branches: supervised learning, unsupervised learning, RL, and application-driven approaches.^16^^,^^17^ Under supervised learning, methods such as classification and regression are employed to predict labeled outputs, with classification specifically highlighted due to its relevance in categorical prediction tasks.^18^ Unsupervised learning includes clustering for pattern discovery and dimensionality reduction for data simplification.^19^ RL represents a distinct paradigm where agents iteratively learn from environmental feedback to optimize decision-making strategies.^20^ Finally, the taxonomy extends to key application domains, including computer vision, natural language processing, and time-series forecasting highlighting the practical implementation of AI/ML techniques across diverse real-world scenarios.^21^ The sections explored the AI/ML integration in nanosensor-enabled ICT systems. This review introduces a holistic synthesis that unifies nanosensors, AI/ML, and ICT architectures, a perspective seldom addressed in the existing literature. It emphasizes AI-driven integration frameworks including edge computing, FL, and hybrid ML as transformative enablers for intelligent, adaptive nanosensor systems. The study also provides structured comparative analyses through detailed tables and figures, summarizing nanosensor types, communication strategies, and ML algorithms while outlining key performance trade-offs. Furthermore, the review identifies critical research gaps, notably the scarcity of nanosensor-specific datasets and the complexities of multimodal data integration, thereby establishing a foundation for advancing standardized, scalable, and data-driven nanosensor-ICT convergence.

## AI/ML integration in nanosensor-enabled ICT systems

**Figure 2:**
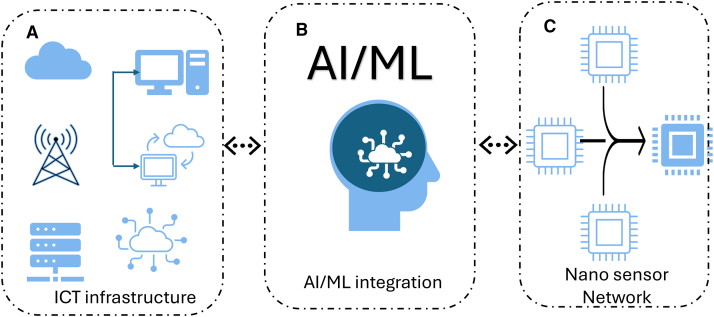
Conceptual architecture of a hardware–software co-designed system integrating multimodal sensing (A) data acquisition through ICT infrastructure, (B) AI/ML-based computing of data obtained from diverse sources, and (C) autonomous edge applications enabled by a Nanosensor Network.

Figure 2 presents a comprehensive architectural framework that delineates the synergistic integration of nanosensor networks, AI/ML algorithms, and ICT infrastructure, forming a dynamic, self-adaptive cyber-physical ecosystem.^22^ At its foundation, the architecture embodies a bidirectional and hierarchical data flow, where nanosensors act as the primary data generators, continuously capturing high-resolution, multimodal signals from complex physical environments. These data streams traverse heterogeneous ICT layers spanning edge nodes, fog gateways, and cloud servers where AI/ML models are strategically deployed based on computational demand, latency constraints, and energy availability. The integration of supervised, unsupervised, and RL algorithms within this layered architecture enables context-aware analytics, predictive modeling, and autonomous decision-making, allowing real-time adaptation of sensing and communication parameters.^23^ The framework operationalizes a closed-loop intelligence cycle, where insights derived from AI inference are propagated back to the nanosensor layer for adaptive calibration, fault detection, and resource optimization.^24^^,^^25^ This feedback mechanism significantly enhances system resilience, energy efficiency, and scalability under dynamically changing environmental or physiological conditions. However, despite its transformative potential, this architecture faces notable technical constraints, including communication latency across multi-tier networks, energy depletion at nanoscale nodes, and data heterogeneity that complicates model convergence. Moreover, maintaining security, privacy, and interoperability across distributed AI-enabled layers remains an unresolved challenge that limits large-scale, real-world deployment. Therefore, while the proposed architecture establishes a conceptually robust and integrative foundation for next-generation intelligent nanosensor systems, its practical realization demands further optimization of lightweight learning models, adaptive communication protocols, and cross-layer security frameworks to ensure reliable, efficient, and sustainable cyber-physical intelligence.^26^

**Figure 3:**
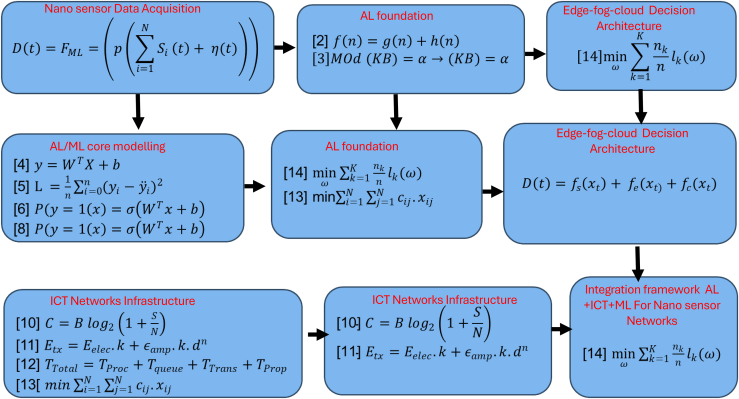
Theoretical linkage of AI/ML integration in nanosensor-enabled ICT systems, showing the relationship between learning types and application domains for intelligent data processing and decision-making

The integration of AI and ML within nanosensor-enabled ICT systems can be formally characterized through the computational models and learning functions presented in the equations in Figure 3, which encapsulate the relationships between sensor data acquisition, feature transformation, decision inference, and adaptive system optimization^27^: Recent studies have increasingly emphasized the need to integrate AI and ML approaches to address persistent limitations in nanosensor performance.^28^ Traditional nanosensor systems often face challenges such as signal noise, baseline drift, and energy constraints, which significantly affect detection accuracy and long-term reliability.^27^ To overcome these issues, researchers have developed AI/ML techniques that enhance signal processing, adaptability, and efficiency. For instance, denoising autoencoders and convolutional neural networks (CNNs) have been applied to reduce random noise and improve feature extraction from complex sensor data.^29^ Similarly, adaptive and online learning algorithms have shown potential in compensating for signal drift by enabling real-time recalibration without manual intervention.^30^ Moreover, lightweight and energy-efficient ML models are being designed to operate on edge devices, minimizing computational load and extending sensor lifespan under constrained power conditions. By linking these nanosensor limitations with targeted AI/ML strategies, the literature demonstrates a growing shift from isolated algorithmic optimization toward holistic, intelligent sensing frameworks. This integration not only strengthens analytical understanding but also paves the way for the development of autonomous and resilient nanosensing systems capable of performing in dynamic and resource-limited environments.^31^

(Equation 1) $$D\left(t\right)=F_{ML}=\left(p\left(\sum_{i=1}^{N}S_{i}\left(t\right)+\eta \left(t\right)\right)\right)$$

Equation 1 models the end-to-end data pipeline, initiating with signal acquisition from an array of nanosensors *S*_*i*_(t), where NNN represents the total number of deployed nanosensors at a given time t. These nanosensors are designed to detect minute variations in physical, chemical, or biological parameters, as mathematically represented in Equation 1. The aggregated signal is affected by noise η(t), which may result from environmental interference, cross-sensitivity, or instrumental drift effects that are more significant at the nanoscale. To ensure that the raw data are usable by AI systems, a preprocessing stage P(⋅) is essential. This stage includes operations such as noise filtering, normalization, feature extraction, and dimensionality reduction, allowing the system to capture meaningful patterns from the data while reducing complexity. The transformed features are then input into an ML model *F*_*ML*_(·), such as neural networks, support vector machines (SVMs), or decision trees.^31^ These models learn from prior data to detect patterns, classify events, and make real-time decisions for adaptive control.^32^ The final output D(t) represents the intelligent decision or prediction made at time t, based on current and past nanosensor data. This dynamic output can be utilized in various smart environments, including health diagnostics, precision agriculture, environmental monitoring, and industrial automation. By integrating nanoscale sensing with AI/ML inface, such ICT systems achieve higher levels of sensitivity, specificity, and adaptability. Recent studies highlight the effectiveness of these models in real-world systems, validating the importance of both signal fidelity and computational intelligence.^32^ Moreover, AI systems rely on fundamental mathematical models. One key example is the A∗ search algorithm, commonly used in pathfinding and graph traversal, defined as *f*(*n*) = *g*(*n*)+*h*(*n*), where *f*(*n*) is the estimated total cost of the path through node *n*, *g*(*n*) is the cost from the start node to *n*, and *h*(*n*) is the heuristic estimate from *n* to the goal (Equation 2). This equation helps AI systems make efficient decisions.^33^^,^^34^ Similarly, knowledge representation in propositional logic applies the model-theoretic notation *Mod*(*KB*) ⊨ *α* ⇒*KB* ⊨ *α*, meaning that if *α* is true in all models of the knowledge base (KB), then *α* is entailed by KB (Equation 3). These formulations form the basis for logical reasoning in AI applications.

(Equation 2) f(n)=g(n)+h(n)

f(n): Estimated total cost of path through node

ng(n): Cost from start node to

nh(n): Heuristic estimate of cost from n to goal

(Equation 3) MOd(KB)=α→(KB)=α

Similarly, ML uses statistical models to learn from data. In linear regression, the output is predicted as *y* = *w*^*T*^*x*+*b*, where *x* represents input features, *w* are the weights, and *b* is the bias term. The Mean Squared Error loss function, $L=\frac{1}{n}\sum {\left(y_{i}-{\hat{y}}_{i}\right)}^{2}$, measures the prediction error (Equation 4). For classification, logistic regression estimates probabilities using the sigmoid function $P\left(y=1|x\right)=\frac{1}{1+e^{-\left(w^{T}x+b\right)}}$. SVMs classify data by maximizing the margin, solving $\min\frac{1}{2}||w|{|}^{2}$ subject to *y*_*i*_(*w*^*T*^*x*_*i*_+*b*) ≥ 1. Neural networks compute outputs through layers using *z* = ∑(*w*_*i*_*x*_*i*_)+*b*, followed by an activation function *a* = *ϕ*(*z*).^34^ RL updates state-action values with Q-learning, $Q\left(s,a\right)\leftarrow Q\left(s,a\right)+\alpha \left[r+\gamma \max_{a^{\prime }}Q\left(s^{\prime },a^{\prime }\right)-Q\left(s,a\right)\right]$, where *γ* is the discount factor, *α* the learning rate, and *r* the reward ((Equation 10), (Equation 11), (Equation 5), (Equation 6), (Equation 7), (Equation 8) and 11).

(Equation 4) $$y=W^{T}X+b$$

X: Input features

W: Weights

b: Bias

y: Output prediction

(Equation 5) $$\text{Loss}\;\text{Function}\;\left(\text{Mean}\;\text{Squared}\;\text{Error}\right):L=\frac{1}{n}\sum_{i=0}^{n}{\left(y_{i}-{\ddot{y}}_{i}\right)}^{2}$$

Logistic Regression (Classification)

(Equation 6) $$P(y=1\left(x\right)=\sigma \left(W^{T}x+b\right)=\frac{1}{1+e^{\left(W^{T}x+b\right)}}$$

SVM

(Equation 7) $$\min_{w,b}\frac{1}{2}{\left\|W\right\|}^{2}\;\text{subject}\;\text{to}\;y_{i}\left(W^{T\;}x_{i}+b\right)\geq 1$$

Neural Networks (Forward Propagation)

For a neuron in a hidden layer:

(Equation 8) $$z=\sum_{i=1}^{n}w_{i}x_{i}+b;a=\emptyset \left(z\right)$$

∅(*z*): Activation function.

RL

(Equation 9) $$Q\left(s,a\right)\leftarrow Q\left(s,a\right)+\alpha [r+\gamma \max_{a}Q\left(s^{ˊ},a^{ˊ}\right)-Q\left(s,a\right)$$

*α*: Learning rate

*γ*: Discount Factor

*r*: Intermediate Reward

*Q*(*s*,*a*): Current State

s,a,sˊ: State, Action, and Next State

In ICT systems, particularly those using nanosensor networks, channel capacity is defined by Shannon’s theorem: C = B log_2_(1 + S/N), where C is the capacity in bits per second, B is bandwidth, and S/N is the signal-to-noise ratio. Wireless sensor energy usage is modeled as: E_tx = E_elec·k + ε_amp·k·d^n^, where E_tx is the energy to send k bits over distance d, E_elec is electronics energy, ε_amp is the amplifier constant, and n is the path loss exponent. Latency in ICT systems is given by: *D*(*t*) = *f*_*s*_(*x*_*t*_)+*f*_*e*_(*x*_*t*_)+*f*_*c*_(*x*_*t*_) Equation 13, summing delays from processing, queuing, transmission, and propagation. Routing optimization problems aim to minimize communication cost: min ΣΣ c_ij·x_ij, where c_ij is the cost between nodes i and j, and x_ij is a binary variable indicating link usage. Based on Shannon’s capacity theorem,^35^

(Equation 10) $$C=B\;\log_{2}\left(1+\frac{S}{N}\right)$$

C: Chanel Capacity.

B: Bandwidth.

Energy consumption in wireless sensor networks (WSNs)

(Equation 11) $$E_{tx}=E_{elec}.k+\epsilon_{amp}.k.d^{n}$$

$E_{tx}$: Energy to transmit k bits over distance d.$E_{elec}$: Energy of Electronic*ϵ*_*amp*_: Amplifier Energy Constantn: Path Loss Exponent

Latency modeling

(Equation 12) $$T_{Total}=T_{Proc}+T_{queue}+T_{Trans}+T_{Prop}$$

$T_{proc}$: Processing delay

$T_{queue}:Queuing\;Delay$

$T_{Trans}:Transmission\;Delay$

$T_{Prop}:Propagation\;Delay$

Routing Optimization (Objective Function)

(Equation 13) $$\min\sum_{i=1}^{N}\sum_{j=1}^{N}c_{ij}.x_{ij}$$

$c_{ij}$: cost of transmission from node i to j

$x_{ij\;}\epsilon \left\{0,1\right\}:Whether\;link\;i\to j\;is\;used$

Integration framework—AL+ICT +ML for nanosensor networks.

FL loss aggregation

(Equation 14) $$\min_{\omega }\sum_{k=1}^{K}\frac{n_{k}}{n}l_{k}\left(\omega \right)$$

$l_{k}$: loss on device k

$n_{k}$: loss on data size

*n*_*k*_: Local data size

Edge Intelligence Decision Function

(Equation 15) $$D\left(t\right)=f_{s}\left(x_{t}\right)+f_{e}(x_{t)}+f_{c}\left(x_{t}\right)$$

*f*_*s*_(*x*_*t*_): On-device sensing inference

$f_{e}(x_{t})$: Edge server inference

$f_{c}\left(x_{t}\right)$: Cloud inference

$x_{t}$: Input data at time t

Integrated systems that combine AI, ML, and ICT utilize FL, edge computing, and hybrid optimization to enhance performance and scalability. FL minimizes global loss across distributed devices as shown in Equation 14: $\min_{\omega }\frac{n_{k}}{n}l_{k}\left(\omega \right)$, where *l*_*k*_ is the local loss at device *k* and *n*_*k*_ is the local data size. Edge intelligence integrates decisions from local, edge, and cloud models as in Equation 15: *D*(*t*) = *f*_*s*_(*x*_*t*_)+*f*_*e*_(*x*_*t*_)+*f*_*c*_(*x*_*t*_), where *x*_*t*_ is the input data at time *t* and *f*_*s*_(*x*_*t*_), *f*_*e*_(*x*_*t*_), and *f*_*c*_(*x*_*t*_) represent the local, edge, and cloud models, respectively. These formulations enable low-latency, energy-efficient, and secure operations, forming the foundation of robust nanosensor-based ICT infrastructures. The convergence of nanosensors, AI/ML algorithms, and ICT technologies allows precision sensing, distributed intelligence, and real-time adaptive decision-making, with nanoscale sensing layers capable of detecting environmental, biological, or chemical parameters with exceptional sensitivity and spatial resolution. The acquired signals, however, are inherently prone to stochastic fluctuations, sensor drift, and environmental interference, which introduce noise and uncertainty into the data stream factors rigorously modeled and quantified in (Equation 1), (Equation 2), (Equation 3), (Equation 4), (Equation 5). As highlighted by Zhang et al.,^36^ advanced signal processing techniques and ML-based denoising strategies are essential to extract meaningful information, ensure reliable data transmission, and maintain system robustness across varying operational conditions.^37^ While nanosensors offer high-resolution detection, their output is inherently noisy, necessitating advanced preprocessing pipelines. These pipelines employ techniques such as Kalman filtering, wavelet decomposition, and dimensionality reduction, which enhance signal-to-noise ratio (SNR) and optimize data structure for ML input.^38^ Upon preprocessing, data enter the ML model FML, wherein supervised or unsupervised algorithms conduct classification, regression, or anomaly detection. Research by Harrou et al.^39^ provides an expansive foundation on deep learning architectures applicable here; however, their effectiveness in low-power, edge environments is limited due to high computational demands. In contrast,^40^ emphasize the importance of class imbalance in sensor data, advocating for balanced training to avoid misclassification, especially in anomaly detection tasks such as fault prediction or pollutant level alerting. A pivotal element of modern intelligent nanosensor systems is FL, which addresses privacy, scalability, and communication overhead. Instead of centralizing raw sensor data, FL aggregates model updates from multiple edge nodes while preserving local data sovereignty^40^^,^^41^. Another research^42^ demonstrated the scalability of FL for real-world applications, albeit with trade-offs in convergence speed and model accuracy. Their work contrasts with traditional cloud-centric AI approaches,^43^ which, while more accurate due to centralized data access, raise significant privacy and latency concerns. To ensure efficiency and feasibility, the underlying ICT framework must be optimized. Shannon’s channel capacity theorem^44^ defines the theoretical upper bound of data throughput, guiding bandwidth allocation in wireless nanosensor networks. Researchers^45^^,^^46^ further developed an energy model detailing the power cost of data transmission based on signal distance and amplification, offering essential insights for energy-harvesting and low-power sensor applications. Nonetheless, their model assumes static topology, which limits its relevance in dynamic, mobile sensor environments. Moreover, latency defined as the total delay from data capture to decision actuation is critically analyzed using the decomposition *T*_*Total*_ = *T*_*Proc*_+*T*_*queue*_+*T*_*Trans*_+*T*_*Prop*_ Equation 12.^47^^,^^48^ This framework identifies queueing delays as a dominant bottleneck in dense sensor networks, prompting the integration of edge intelligence. Edge intelligence further augments this architecture via a distributed decision function *D*(*t*) = *f*_*s*_(*x*_*t*_)+*f*_*e*_(*x*_*t*_)+*f*_*c*_(*x*_*t*_), blending local, edge, and cloud-based inference. Compared with centralized AI systems,^49^^,^^50^ this model enhances latency reduction and real-time responsiveness, albeit requiring coordination protocols to ensure model consistency and accuracy across layers. The hierarchical model provides resilience, enabling immediate local decisions while utilizing cloud-level resources for deep insights and long-term planning. Lastly, intelligent reasoning and optimization leverage symbolic AI and classical planning models.^51^ A∗ pathfinding algorithms and propositional logic support goal-driven decision-making, while linear models and neural networks provide probabilistic inference. However, the limitations of symbolic AI such as inflexibility and brittleness in unstructured environments necessitate hybrid approaches combining symbolic reasoning with neural learning, an area still in active research.^52^ In the context of edge AI, recent studies have demonstrated that deploying models at the edge can reduce latency from approximately 150 ms to below 50 ms. Additionally, offloading computations to low-power microcontrollers has been shown to decrease energy consumption by up to 40%.^53^^,^^54^^,^^55^^,^^56^^,^^57^ These findings provide further support for the technical and performance advantages of edge deployment, enhancing the analytical depth of our discussion. Overall, while the integration of nanosensors with AI/ML and ICT infrastructures demonstrates immense potential, several critical challenges remain. Traditional models, though foundational, often struggle to handle the dynamic, high-dimensional, and privacy-sensitive nature of nanosensor data. Similarly, FL and edge intelligence, despite their promise, require further improvement in algorithmic efficiency, hardware compatibility, and real-world implementation. A comparative synthesis of recent studies highlights the need for adaptive, scalable, and privacy-preserving architectures to fully exploit the capabilities of AI-enhanced nanosensing. The following section provides a general overview of recent efforts in integrating nanosensor technologies within ICT systems.

## Overview of nanosensor technologies in ICT

Following the theoretical feasibility analysis conducted in this study, the integration of nanotechnology with ICT has fundamentally redefined the mechanisms through which data are acquired, processed, and utilized across diverse sectors. At the nanoscale, sensor arrays can enable ultra-sensitive, real-time detection of physical, chemical, or biological stimuli, while their integration with ICT infrastructures facilitates high-throughput data transmission, edge-level processing, and intelligent decision-making. This convergence enables the deployment of distributed nanosensor networks capable of *in situ* analytics, autonomous operation under constrained energy and computational resources, and dynamic adaptation to environmental stimuli through AI-optimized communication protocols and embedded processing architectures.^58^ Nanosensor technologies, defined by their ability to operate at the molecular or atomic level, offer ultra-sensitive, real-time detection capabilities.^59^ However, while their potential is vast, the integration of nanosensors into existing ICT infrastructure presents both engineering opportunities and substantial scientific challenges, especially in terms of reliability, standardization, and scalability. Nanosensors are classified into various types based on their specific applications and generally exhibit superior sensitivity and selectivity toward target analytes or environmental conditions compared with conventional sensors. However, like all technologies, they possess both distinct advantages and inherent limitations, which are discussed in detail in the following section.

## Types of nanosensors: Capabilities and limitations

Nanosensors exhibit a high degree of functional diversity, typically classified by their transduction mechanisms such as optical, electrochemical, or piezoelectric—and target detection modalities, including chemical, biological, and environmental analytes. Their enhanced performance arises from nanoscale effects like quantum confinement and high surface-to-volume ratios, enabling highly sensitive and selective detection. Chemical nanosensors, particularly those based on carbon nanotubes and metal oxide nanoparticles, have demonstrated exceptional sensitivity to volatile organic compounds and environmental pollutants.^60^ However, issues such as baseline drift, cross-sensitivity to non-target gases, and long-term stability remain unresolved.^61^ Biosensors utilizing nanostructured transducers are central to precision diagnostics. Gold nanoparticles and graphene-based platforms have enabled low detection limits and enhanced selectivity.^62^ Yet, the reproducibility of such biosensors at the point-of-care scale is hampered by challenges in biointerface engineering and surface functionalization consistency.^63^ Optical nanosensors provide compelling solutions for label-free and multiplexed analysis, employing phenomena such as surface plasmon resonance (SPR) and Förster resonance energy transfer (FRET).^64^ Despite their demonstrated efficacy under controlled laboratory conditions, the integration of AI with nanosensor systems in real-world environments presents a set of unique and persistent challenges. In complex, dynamic media, such as biological fluids, soil, or urban atmospheres, signal attenuation, photobleaching, and nonlinear environmental interactions can significantly degrade sensor performance and data reliability. These effects not only compromise detection sensitivity but also introduce variability that complicates real-time AI-driven interpretation. Furthermore, the heterogeneity of data from nanosensors operating under diverse conditions demands advanced AI models capable of robust generalization and adaptive learning. Ensuring synchronization between nanoscale signal acquisition and macroscopic decision-making, while maintaining low power consumption, minimal latency, and high fidelity, remains a critical bottleneck. Overcoming these hurdles requires innovations in AI model robustness, noise-aware signal processing, and context-adaptive learning algorithms that can function reliably in the presence of physical constraints inherent to nanoscale sensing.^65^ Physical nanosensors, notably nano-electromechanical systems, are adept at measuring stress, pressure, or temperature with unprecedented precision.^66^ However, their inherent mechanical fragility and vulnerability to environmental noise present major challenges for reliable field deployment, especially in dynamic outdoor environments or *in vivo* biomedical applications where structural stability and signal fidelity are critical.^67^ The miniaturization to the nanoscale does not inherently guarantee superiority. Instead, it necessitates precise material control, robust calibration protocols, and advanced signal processing frameworks to ensure accuracy and utility across varied deployment scenarios.^68^

## Communication technologies: Bridging the nano-to-macro gap

While the previous section discussed the functional classifications, performance advantages, and limitations of nanosensors across various applications, their effective integration into real-world systems demands more than advanced sensing capabilities. One of the key challenges lies in ensuring reliable and efficient data transmission from the nanoscale domain to macroscopic ICT systems. The following subsection explores Communication Technologies that address this challenge by bridging the nano-to-macro gap, ensuring that sensor data are efficiently captured, transmitted, and utilized in broader networks. The data output of nanosensors must be efficiently communicated, interpreted, and acted upon a task that demands equally advanced communication technologies.^69^ Conventional wireless protocols like ZigBee and Bluetooth Low Energy offer low-power operation and are suitable for wearable or indoor nanosensor applications.^70^ However, their limited data throughput and short-range capabilities constrain broader deployments, especially in smart cities or precision agriculture.^71^ LoRa (Long Range) technology has emerged as a promising alternative, offering extended coverage with minimal energy consumption.^72^ While ideal for sporadic data transmission from nanosensor nodes in remote areas, its low bandwidth makes it unsuitable for high-frequency or image-based sensing.^73^ Emerging Terahertz (THz) communications represent a critical leap forward, especially in nano-networks where data rates and node density are exceptionally high.^74^ Nevertheless, THz systems currently face formidable barriers in terms of signal propagation loss, antenna miniaturization, and channel modeling.^75^ Additionally, the required nanophotonic and plasmonic components are still in the experimental stage, limiting practical implementations.^76^ Nano-communication frameworks, particularly molecular and plasmonic communication models, are gaining traction for in-body applications such as drug delivery and neural interfacing.^77^ These systems are biocompatible and energy efficient but are far from maturity.^78^ The lack of standardized architectures, synchronization issues, and high latency in molecular propagation make them suitable only for niche applications at present.^79^ The overarching limitation remains the absence of a unified protocol stack or interoperability model that bridges nanoscale networks with conventional ICT layers. Without this, the vision of seamless nano-macro integration will remain theoretical.^80^^,^^81^ Building on the discussion of nanosensor classifications and their communication frameworks bridging the nano-to-macro interface, it becomes evident that the true potential of these technologies is realized through their integration within broader ICT ecosystems. The synergy between nanoscale sensing and advanced ICT infrastructure enables real-time monitoring, intelligent decision-making, and adaptive system responses across various domains. The following section delves into the applications of ICT, emphasizing its transformative impact, integration strategies with nanosensor networks, and the emerging prospects that define the next frontier of smart and connected systems.

## ICT applications: Impact, integration, and future prospects

The application landscape of nanosensors within ICT is broad and multidisciplinary, yet practical deployments remain uneven across sectors.^82^ In healthcare, nanosensors embedded in wearable systems have transformed real-time monitoring of biomarkers such as glucose, lactate, and cortisol.^83^ Despite this potential, few have achieved widespread clinical adoption due to biosafety concerns, calibration complexity, data privacy issues, and stringent regulatory requirements.^84^ In agriculture, nanosensors enable detailed soil health analysis, pest detection, and crop phenotyping.^85^ When integrated with IoT platforms, they support data-driven precision agriculture.^86^ However, challenges such as economic feasibility, device durability in harsh environments, data ownership, and cybersecurity risks persist, particularly in developing regions.^87^ Environmental monitoring has advanced significantly with nanosensors capable of detecting heavy metals, pathogens, and greenhouse gases at ultra-trace concentrations.^88^ Nevertheless, large-scale deployment remains limited due to high production costs, device fouling, and insufficient energy autonomy.^89^ In smart infrastructure, embedded nanosensors are increasingly used for structural health monitoring of bridges, tunnels, and buildings, providing early warnings of stress or fatigue. Yet, integrating these sensors into existing structures while maintaining long-term energy supply, data fidelity, and network security remains a major challenge.^90^ Beyond technical barriers, non-technical factors such as regulatory uncertainty, data security, privacy in nanoscale communication, and ethical implications of large-scale sensor deployments are critical concerns. Addressing these requires robust governance frameworks and interdisciplinary collaboration among experts in nanofabrication, systems engineering, data analytics, and cyber-physical systems design. Moreover, evolving regulatory and ethical guidelines are essential to ensure the safe, equitable, and sustainable integration of nanosensor technologies within ICT infrastructures.^91^^,^^92^

Table 1 delineates the progressive diversification of nanosensor deployment across biomedical, environmental, and industrial domains, emphasizing the interplay between material design, detection principles, and communication architectures. Chemiresistive nanosensors, particularly those based on metal oxide semiconductors and carbon nanotube (CNT) polymer composites, have gained prominence due to their superior sensitivity and molecular selectivity arising from quantum confinement effects and tunable surface functionalization.^66^^,^^67^ The integration of these nanosensors with low-power communication protocols such as radiofrequency identification (RFID), Wi-Fi, and Zigbee facilitates real-time, distributed monitoring frameworks with enhanced scalability and minimal energy expenditure, enabling continuous data acquisition in dynamic field conditions.^68^^,^^69^ Nevertheless, despite their functional versatility, these systems face persistent limitations associated with signal instability under fluctuating environmental conditions, susceptibility to electromagnetic interference, and degradation of surface-active sites over time.^70^ Such challenges highlight the necessity for the incorporation of intelligent compensation mechanisms such as adaptive calibration, noise-aware filtering, and temperature-humidity compensation algorithms within the sensing and communication pipeline.^71^^,^^72^ Addressing these issues is crucial for maintaining signal integrity, improving cross-sensor interoperability, and ensuring reliable, long-term deployment in safety-critical and high-precision applications.^73^ In biomedical diagnostics, nanosensors have shown exceptional promise due to their ability to provide high selectivity and sensitivity toward disease biomarkers. For instance, aptamer-based biosensors, enzyme-functionalized gold nanoparticles, and antibody-coated Au nanoparticles utilize mechanisms such as electrochemical sensing, catalytic reactions, and SPR to enable early-stage disease detection. These devices, when integrated with optical signaling or fiber-optic links, facilitate non-invasive, real-time monitoring, which is crucial for applications in diabetes management, cancer detection, and genetic screening. Nevertheless, challenges such as biofouling, short operational lifespan, and matrix interference in complex biological fluids continue to hinder their widespread clinical translation, highlighting the need for further material optimization and robust anti-fouling strategies. Physical and chemical sensing applications further demonstrate the versatility of nanomaterials and detection mechanisms. Quantum dot-based fluorescence sensors for pH sensing, ZnO nanowire piezoelectric sensors for pressure,^64^ and ruthenium-based phosphorescence quenching sensors for oxygen monitoring are indicative of the fine-tuned material-signal transduction pairings made possible by nanoscale engineering.^74^ These sensors, integrated with fluorescent, capacitive, and luminescent communication channels, are particularly effective for intracellular monitoring, wearable devices, and respiratory diagnostics. Yet, issues such as photobleaching, sensitivity to ambient conditions, and the need for consistent calibration underscore the importance of improving both stability and selectivity for reliable real-time sensing in physiological environments. Lastly, the integration of nanosensors in agricultural, industrial, and defense domains showcases their adaptability and utility in high-impact fields. Applications such as soil nutrient monitoring using ion-selective electrode-based nanosensors,^54^ toxic gas leakage detection with Pd-decorated graphene nanosensors,^55^ and explosive detection via nanowire field-effect transistors exemplify the transition from lab-scale prototypes to field-deployable technologies. Exploring advanced communication protocols such as LoRaWAN and Zigbee, nanosensor networks demonstrate the capability for long-range, low-power, and reliable data transmission, an essential feature for precision agriculture, industrial safety, and real-time environmental security applications.^75^ These protocols offer high scalability and low-energy footprints, which are particularly advantageous for energy-constrained nanoscale devices that rely on limited power sources.^76^ Nevertheless, several critical challenges remain unresolved. Nanosensors deployed in harsh or variable environments face degradation in sensing accuracy and communication stability due to mechanical stress, temperature fluctuations, and electromagnetic interference.^77^ Furthermore, issues related to sensor ruggedization, reusability, and large-scale cost-effective manufacturing continue to hinder their widespread industrial adoption.^78^ Overcoming these barriers requires a convergence of multidisciplinary innovations advances in nanofabrication techniques; the development of energy-harvesting mechanisms such as piezoelectric, thermoelectric, and Random Forest (RF)-based power recovery; and the design of adaptive, fault-tolerant communication architectures capable of supporting edge intelligence and self-organizing behavior.^79^^,^^80^ Achieving these developments will be pivotal in establishing fully autonomous, resilient, and scalable nanosensor networks for next-generation smart environments.^81^

**Table 1:** Classification of nanosensors based on functionality and communication techniques

Functionality	Type of Nanosensor	Detection Mechanism	Communication Technique	Application Domain	Reference
Gases detection	Metal oxide nanosensors	Chemiresistive	Electromagnetic (RFID/Wi-Fi)	Environmental monitoring	Chaturvedi et al.^89^
Biomarker	Aptamer-based biosensors	Electrochemical	Optical signaling	Biomedical diagnostics	Tripathy et al.^93^
pH sensing	Quantum dot nanosensors	Fluorescence	Fluorescent modulation	Intracellular monitoring	Islam et al. ^94^
Temperature and humidity sensing	CNT-based thermal nanosensors	Thermoelectric effect	Electromagnetic (Infrared (IR), Radio Frequency (RF))	Nanoelectronics, health monitoring	Huang et al.^95^
Glucose sensing	Enzyme-functionalized gold nanosensors	Catalytic reaction	Electrochemical signal	Diabetes management	Zhu et al.,^96^
DNA/RNA detection	Graphene oxide nanosensors	FRET (fluorescence resonance)	Optical nanocommunication	Genetic screening	Li et al.^97^
Pathogen detection (*E. coli*)	Magnetic nanoparticle biosensors	Magnetic field variation	Magnetic coupling	Food safety, healthcare	Xiong et al.^98^
Pressure sensing	ZnO nanowire piezoelectric sensors	Piezoelectric response	Capacitive/Resistive	Wearable devices, robotics	Liu et al.^99^
Drug delivery and monitoring	Mesoporous silica nanosensors	Controlled release detection	Bioluminescent signal	Targeted therapy	Pancino et al.^29^
Volatile Organic Compound (VOC) sensing	Polymer-coated CNT sensors	Chemiresistive	Wireless signal (Zigbee/Wi-Fi)	Industrial safety	Hemdan et al.^100^
Humidity detection	Graphene oxide humidity sensors	Capacitance change	Capacitive communication	Environmental sensors	Mim et al.^101^
Explosive detection	Nanowire field-effect transistor sensors	Field-effect modulation	Electrical signal transmission	Defense and security	Jalalvand et al.^102^
Heavy metal ion detection	Functionalized silica nanoparticles	Surface adsorption spectroscopy	Optical nanocommunication	Water purification	Darwish et al.^103^
Cancer biomarker detection	Antibody-functionalized Au nanoparticles (NPs)	SPR (Surface Plasmon Resonance)	Optical fiber link	Early cancer diagnostics	Jang et al.^104^
Chemical monitoring	Fluorescent nanosensors	Ion-sensitive fluorophores	Fluorescent	Neuroscience	Raghu et al.^105^
Radiation detection	Scintillating nanoparticle sensors	Radioluminescence	Optical readout	Nuclear, medical imaging	Boruah et al.^93^
Strain sensing	CNT/polymer nanocomposites	Piezoresistive effect	Wireless strain transmission	Structural health monitoring	Rawtani et al.^106^
Oxygen level monitoring	Ruthenium-based nanosensors	Phosphorescence quenching	Luminescence-based signaling	Respiratory diagnostics	Ghosh et al.^107^
Real-time sweat analysis	Wearable flexible nanosensors	Electrochemical sensing	Bluetooth/IoT integration	Sports medicine, health monitoring	Ghorbian et al.^108^
Smart farming (soil nutrient sensing)	Nanosensors embedded in soil	Ion-selective electrodes	Wireless communication (LoRa)	Precision agriculture	M. Sharma et al.^109^
Toxic gas leakage detection	Pd-decorated graphene nanosensors	Conductance modulation	Zigbee/LoRaWAN	Industrial safety	Siqi et al.^110^
Neurotransmitter detection	Carbon quantum dot sensors	Fluorescence or electrochemical	Wireless or optical signaling	Neurological research	Kulkarni et al.^111^

**Figure 4:**
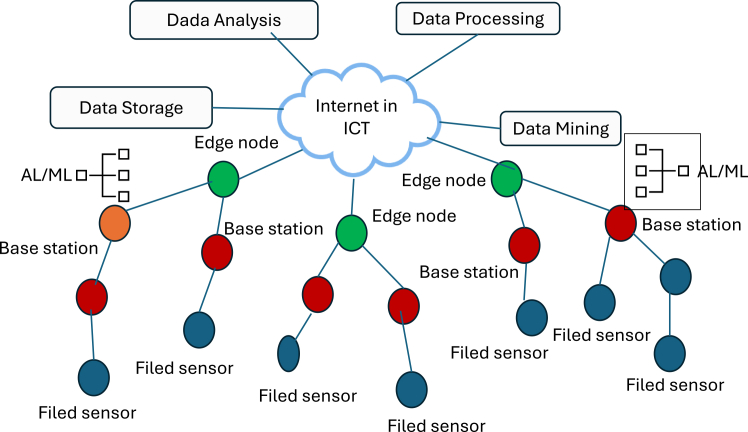
Architecture of a Typical Nanosensor Network with ICT Components

Figure 4 presents a hierarchical architecture integrating sensors, base stations, and cloud-based ICT functions. Sensors collect raw data, which are aggregated and transmitted via base stations to the cloud. ICT layers handle data processing, mining, analysis, and storage. While efficient, challenges remain in latency, interoperability, and real-time scalability.^95^^,^^112^^,^^113^^,^^114^ Recent advances in nano-enabled Internet of Things (IoT) platforms have led to the emergence of smart nanosensor networks capable of supporting a wide range of applications, from biomedical diagnostics and environmental monitoring to industrial automation.^96^^,^^115^ It further illustrates a cloud-based hierarchical architecture designed to manage, process, and analyze data generated by these nanosensors.^93^^,^^98^^,^^99^^,^^100^^,^^101^^,^^102^^,^^103^^,^^104^^,^^105^^,^^106^^,^^107^^,^^108^^,^^116^^,^^117^^,^^118^^,^^119^ This architecture underscores the essential components and data flow pathways that facilitate communication between nanosensors, aggregation layers (base stations), and cloud-based computational resources.^120^ At the heart of this model lies a centralized cloud infrastructure, which integrates diverse services including data analysis, processing, mining, and storage.^109^ This centralized model offers scalability, flexibility, and support for computationally intensive tasks such as ML-based anomaly detection and predictive diagnostics. However, this architecture also introduces potential bottlenecks related to latency, bandwidth constraints, and cloud dependency especially in time-critical domains like neurophysiological monitoring or automated surgical interventions.^110^ The lower layers of the architecture represent the distributed nanosensor nodes (depicted in light blue), which serve as the primary data acquisition units. These nanosensors are linked to intermediate aggregation points or base stations (green), which handle local preprocessing, protocol conversion, and data buffering. This intermediate layer is particularly vital for reducing energy consumption, minimizing data redundancy, and managing communication overhead.^111^ Nonetheless, the current architecture does not elaborate on key operational challenges such as data synchronization, collision avoidance, or energy-harvesting strategies, which are critical for sustaining long-term deployments in energy-constrained environments.^121^ The upper architectural layer focuses on advanced computational functionalities hosted in the cloud. Data analysis modules support feature extraction and classification, while data mining mechanisms uncover hidden correlations across spatial and temporal datasets. Data storage ensures historical data archiving for longitudinal studies.^122^ Despite these advantages, the absence of edge or fog computing paradigms limits real-time decision-making and local autonomy. For high-throughput applications such as real-time sweat analysis or toxic gas detection in industrial settings, edge computing can offer improved responsiveness, reduced network congestion, and enhanced fault tolerance. While the figure offers a clear visualization of system functionality and modular interactions, it overlooks several practical considerations.^123^ These include cybersecurity protocols, encryption schemes, and interoperability standards, which are fundamental to ensuring secure, seamless operation across heterogeneous IoT platforms.^124^ Moreover, to improve the resiliency and adaptability of nanosensor networks, future architectures should incorporate blockchain-based data integrity mechanisms, AI-powered edge nodes, and self-healing network capabilities. Addressing these issues will significantly enhance the robustness and applicability of nanosensor networks in next-generation IoT ecosystems.^125^ As nanosensors become increasingly embedded within ICT infrastructures and communication technologies continue to evolve, the complexity and volume of data generated at the nanoscale necessitate advanced analytical approaches. ML has emerged as a pivotal enabler in this context, offering powerful tools for pattern recognition, anomaly detection, predictive modeling, and system optimization. The following section explores the role of ML in nanosensor systems, highlighting how data-driven intelligence enhances the performance, adaptability, and scalability of nanosensor-based applications across diverse fields.

## Role of machine learning in nanosensor systems

The integration of ML into nanosensor systems has catalyzed a transition from static sensing platforms to intelligent, self-adaptive systems capable of learning, interpreting, and acting upon complex environmental or physiological signals. This synergy is not merely a computational enhancement, it represents a fundamental shift in how nanoscale sensing architectures interface with dynamic, uncertain real-world conditions. Nevertheless, while ML offers substantial promises, its practical deployment in nanosensor-based frameworks is still constrained by data scarcity, power limitations, and interpretability challenges. Within the broader application of ML in nanosensor systems, supervised learning models play a central role in enabling predictive analytics by learning from labeled datasets.^126^ These models ranging from decision trees to deep neural networks are instrumental in identifying complex patterns and making accurate inferences based on sensor data. However, despite their predictive power, they often face practical constraints such as data labeling requirements, overfitting risks, and computational costs. The following section critically examines supervised learning models, weighing their strengths in predictive accuracy against the limitations and trade-offs encountered in real-world nanosensor applications.

## Supervised learning models: Predictive accuracy vs. practical constraints

Supervised learning models remain the most widely applied ML category in nanosensor applications, largely due to their predictive reliability when trained on labeled datasets.^127^ SVMs have demonstrated excellent performance in classifying biosensor outputs, such as cancer biomarkers and bacterial strains, owing to their capacity to handle high-dimensional feature spaces.^10^ However, the model’s sensitivity to kernel parameters and limited scalability under large-scale datasets constrains its broader applicability, especially in streaming sensor data. RFs offer improved robustness through ensemble learning and feature importance metrics, making them particularly useful in multi-parameter chemical sensing and environmental monitoring. Yet, RFs can be computationally expensive, with memory demands and inference time increasing with the number of estimators.^128^ k-Nearest Neighbors (k-NNs), despite its conceptual simplicity and non-parametric nature, performs poorly in nanosensor networks due to its intensive computation at inference and susceptibility to noisy data a common feature in low-power or harsh-environment nanosensing.^129^ Artificial Neural Networks (ANNs), known for their ability to model complex, nonlinear input-output relationships, have been effectively applied to chemical vapor detection, Volatile Organic Compound (VOC) profiling, and multimodal biosignal interpretation.^130^ However, in practice, they suffer from overfitting in small-sample conditions, a prevalent issue in nanosensor domains where large, annotated datasets are difficult to obtain.^131^ In short, while supervised learning enables robust classification and regression, its dependency on high-quality labeled datasets and compute-heavy training regimes makes it less suitable for resource-constrained or decentralized nanosensor deployments.^132^ While supervised learning offers high predictive accuracy, its dependence on labeled datasets poses challenges, particularly in nanosensor environments where annotated data are often scarce or costly to obtain. In such cases, unsupervised learning provides a compelling alternative by uncovering hidden patterns, clusters, and structures within unlabeled data. This capability is especially valuable for anomaly detection, feature extraction, and exploratory data analysis in dynamic or data-sparse nanosensor systems. The following section explores the exploratory power of unsupervised learning, emphasizing its strengths and limitations in enabling autonomous insights where labeled data are minimal or unavailable.

## Unsupervised learning: exploratory power in low-labeled environments

In contrast to supervised approaches, unsupervised learning models address the challenge of unlabeled or sparsely labeled data, an inherent condition in real-time, *in situ* nanosensor operation.^133^ K-means clustering has been widely applied to analyze sensor outputs in environmental monitoring and spatial mapping, owing to its algorithmic simplicity, scalability, and low computational overhead. Despite its effectiveness in partitioning high-dimensional sensor data, it assumes spherical clusters and equal variance, which may limit accuracy in heterogeneous sensing environments.^97^ Nevertheless, its assumption of isotropic cluster geometry and sensitivity to initialization often leads to poor clustering in heterogeneous or high-noise data.^16^ DBSCAN overcomes some of these limitations by identifying clusters of arbitrary shape and handling noise more effectively, an essential feature when working with chemically unstable or biofouling-prone sensors.^133^ Yet, its effectiveness is hindered by its sensitivity to the epsilon parameter, particularly in unevenly distributed datasets. Principal Component Analysis (PCA) serves as a powerful tool for feature dimensionality reduction, particularly for high-dimensional outputs like hyperspectral sensor data or multiplexed bioassays.^134^ While PCA enhances model efficiency and mitigates overfitting, its assumption of linearity often results in loss of information critical for interpreting nonlinear nanoscale interactions. Overall, unsupervised learning offers critical value in data exploration and anomaly detection but requires post hoc interpretation mechanisms to extract actionable insights—a step often neglected in real-world implementations. Beyond supervised and unsupervised approaches, RL and DRL offer powerful frameworks for enabling nanosensor systems to learn optimal actions through interaction with dynamic environments.^135^^,^^136^ These models support continuous adaptation, decision-making under uncertainty, and long-term performance optimization critical capabilities for autonomous and responsive nanosystems. The following section examines how RL and DRL contribute to the development of adaptive nanosensor systems, focusing on their learning paradigms, application potential, and implementation challenges.

## Reinforcement and deep reinforcement learning: toward adaptive nanosystems

RL offers a fundamentally different paradigm centered on sequential decision-making and continuous policy optimization through interaction with dynamic environments. This framework aligns well with the operational demands of intelligent nanosensor systems, enabling autonomous adaptation to non-stationary conditions, real-time resource allocation, and long-term performance optimization under uncertainty. Applications range from adaptive sampling strategies in mobile sensor swarms to real-time feedback control in biosensing and drug delivery. Despite this alignment, RL methods suffer from significant limitations, including sample inefficiency, slow convergence, and vulnerability to non-stationary conditions, challenges that are magnified in nanoscale platforms with limited memory and computational throughput. DRL extends this paradigm by integrating neural networks to approximate high-dimensional policies and value functions, enabling complex behavior learning in autonomous nano systems. For example, DRL has been explored for adaptive thresholding in wearable biosensors and environmental optimization in closed-loop sensing systems. However, DRL models are prone to instability during training and often require extensive hyperparameter tuning. Their black-box nature also raises concerns about interpretability and safety, especially in biomedical or critical infrastructure applications.^67^^,^^72^^,^^86^^,^^137^ While individual ML paradigms supervised, unsupervised, and RL offer distinct advantages, real-world nanosensor systems often require more resilient and generalizable solutions. Hybrid and ensemble approaches address this need by combining multiple models or learning strategies to leverage their complementary strengths and mitigate individual weaknesses. This model fusion enhances predictive robustness, adaptability, and fault tolerance in complex and dynamic sensing environments. The following section explores how hybrid and ensemble methods contribute to robust nanosensor intelligence, emphasizing their architectures, integration strategies, and performance benefits.

## Hybrid and ensemble approaches: robustness through model fusion

Hybrid and ensemble ML models offer promising pathways to overcome the limitations of individual algorithms, especially when dealing with the multidimensional, heterogeneous, and noisy data characteristic of nanosensors. CNN-Recurrent Neural Network (RNN) hybrids have been employed to capture both spatial and temporal dynamics in nanosensor time-series data, demonstrating high accuracy in applications such as physiological monitoring and speech-driven environmental sensing. Ensemble models, such as XGBoost or Stacked Generalization Frameworks, enhance prediction robustness and reduce model variance. In nanosensor systems, they have shown superior performance in multi-class chemical detection and composite material degradation monitoring. However, these approaches often introduce significant computational overhead, which may be incompatible with the real-time constraints and energy budgets of embedded nanosensor platforms.^138^^,^^139^ Moreover, the deployment of hybrid models requires careful tuning of fusion strategies (early vs. late fusion) and often suffers from reduced model interpretability an important concern in regulated sectors such as healthcare and environmental compliance.^140^ ML has undoubtedly enhanced the intelligence and functional capacity of nanosensor systems. However, true realization of autonomous nanosensing networks requires more than high accuracy; it demands interpretability, adaptability, and computational frugality. A future-proof ML-nanosensor integration strategy must therefore address not only algorithmic performance but also embedded system constraints, ethical deployment, data privacy, and cross-domain standardization.

The integration of ML algorithms into nanosensor networks has significantly advanced intelligent sensing platforms across biomedical, environmental, and industrial domains. Table 2 provides a structured comparison of major ML paradigms—supervised, unsupervised, reinforcement, hybrid, and ensemble learning—detailing their application areas, performance metrics, and inherent constraints. Supervised algorithms such as SVMs, RFs, k-NNs, and ANNs have achieved strong predictive accuracy across various nanosensor applications; for example, SVMs have attained 90–98% accuracy in cancer diagnostics involving high-dimensional datasets. However, these conventional models exhibit critical limitations when deployed in nanosensor environments characterized by energy scarcity, limited on-node computation, and non-stationary data streams. Their reliance on large, well-labeled datasets and intensive hyperparameter tuning often leads to overfitting, degraded generalization, and excessive power consumption under real-time nanosensor constraints. Moreover, the lack of adaptability to dynamic sensing conditions and insufficient model interpretability hinder their deployment in safety-critical biomedical and environmental monitoring scenarios. These challenges underscore the necessity for lightweight, noise-aware, and resource-adaptive ML architectures specifically optimized for nanosensor-based intelligent systems.^149^ Despite this, their computational intensity often renders them unsuitable for real-time or embedded applications. RFs, frequently applied in gas sensor networks, resist overfitting and strong generalization capabilities. However, their performance can degrade with poorly curated or imbalanced datasets. k-NN remains a favored choice for microbial sensing due to its simplicity and non-parametric nature but suffers from latency and scalability issues as data volume increases. ANNs, with their capacity to learn complex, nonlinear mappings, have proven valuable in chemical pattern analysis, delivering accuracies around 96%.^150^ Nevertheless, these models present inherent challenges related to overfitting, hyperparameter sensitivity, and limited interpretability, which become increasingly critical under low-sample, high-noise nanosensor conditions where data variability and signal drift are significant. Unsupervised learning algorithms, particularly K-Means and DBSCAN, have been employed for clustering and anomaly detection within nanosensor and chemical data contexts. While K-Means remains popular due to its computational efficiency and scalability, its reliance on predefined cluster numbers and sensitivity to initial centroid selection often lead to suboptimal convergence and poor robustness in nonlinearly separable or high-dimensional feature spaces. In contrast, DBSCAN offers improved performance in detecting irregular cluster boundaries and noise points, yet it is constrained by the difficulty of parameter selection (ε, MinPts) and its declining effectiveness in high-dimensional nanosensor datasets. These limitations underscore the need for adaptive and hybrid unsupervised learning frameworks capable of dynamically tuning parameters and preserving data structure fidelity in complex nanosensor environments.^101^ DBSCAN, conversely, is well suited for applications like gas leak detection due to its robustness to noise and ability to identify arbitrarily shaped clusters. However, its performance is contingent on precise parameter selection, particularly the epsilon radius and minimum point threshold. Dimensionality reduction techniques such as PCA play a pivotal role in feature reduction and noise filtration. PCA has demonstrated around 95% effectiveness in enhancing classifier performance by eliminating redundant data. Its main drawback lies in its linear assumptions, which can underperform in nonlinear sensor datasets common in biomedical and chemical domains. RL methods like Deep Q-Learning and Deep Deterministic Policy Gradient (DDPG) are gaining traction for adaptive sensing and control tasks in dynamic environments. These models excel at learning delayed reward strategies and optimizing sensor actuation policies.^94^ However, their high computational demand and sample inefficiency restrict their deployment on edge or low-power IoT nodes. Hybrid models such as the CNN-RNN combination are increasingly applied to wearable sensor systems to exploit both spatial and temporal correlations. With a reported performance of 97%, these models offer state-of-the-art accuracy in activity recognition and physiological monitoring.^142^ However, the associated inference costs and memory footprints can pose constraints for deployment in embedded nanosystems. Finally, ensemble learning algorithms such as XGBoost stand out in multi-class nanosensor applications like chemical compound detection, where they exhibit superior accuracy (98–99%) and resilience to missing data. The trade-off lies in their computational and memory expense, which requires efficient model compression for resource-limited platforms. In summary, while each algorithm offers distinct advantages tailored to specific application needs, there is a growing need for adaptive, hybridized approaches that integrate the strengths of multiple models while minimizing their weaknesses. Future directions should emphasize energy-aware learning, online model updating, and explainable AI to ensure reliability, interpretability, and real-world viability in nanosensor-driven smart systems.

**Table 2:** Summary of ML algorithms used in Nanosensor applications with performance outcomes

ML Algorithm	Category	Application in real application	Strengths	Limitations	Reported Performance	Reference
Support Vector Machine	Supervised	Cancer diagnostics by measuring molecular concentration	Handles high-dimensional data	Slow	90–98%	Thikra et al.^119^
Random Forest	Supervised	Gas sensors by measuring gas concentration	Reduces overfitting	Poor data handling	95%	Sun et al.^141^
k-Nearest Neighbors	Supervised	Microbial sensing by measuring gram +ve and gram -ve	Easy to implement	Slow	85–92%	Princz et al.^22^
Artificial Neural Network	Supervised	Chemical pattern analysis by identifying chemical content	Learns nonlinear mappings; flexible	Risk of overfitting	96%	Carone et al.^142^
K-Means	Unsupervised	Chemical clustering by mapping compound	Easy to implement	Sensitive to initialization	80–90%	Arjun et al.^143^
DBSCAN	Unsupervised	Gas leak detection by gas component	Robust to noise	Low sensitivity	86%	Alimisis et al.^144^
Principal Component Analysis	Unsupervised	Feature reduction for data quantification	Noise filtering	Captures only linear variance	95%	Kim et al.^145^
Deep Q-Learning/DDPG	Reinforcement	Adaptive sensing for heavy data classification	Learns complex behavior; supports delayed rewards	High computation requirements	89%	Wang et al.^146^
CNN-RNN Hybrid	Hybrid	Wearable systems for health determination	Captures spatial and temporal features	High inference cost	97%	Jouini et al.^147^
XGBoost	Ensemble	Multi-class chemical detection by chemical component sensing	High accuracy; handles missing data	Expensive	98–99%	Godwin et al.^148^

**Figure 5:**
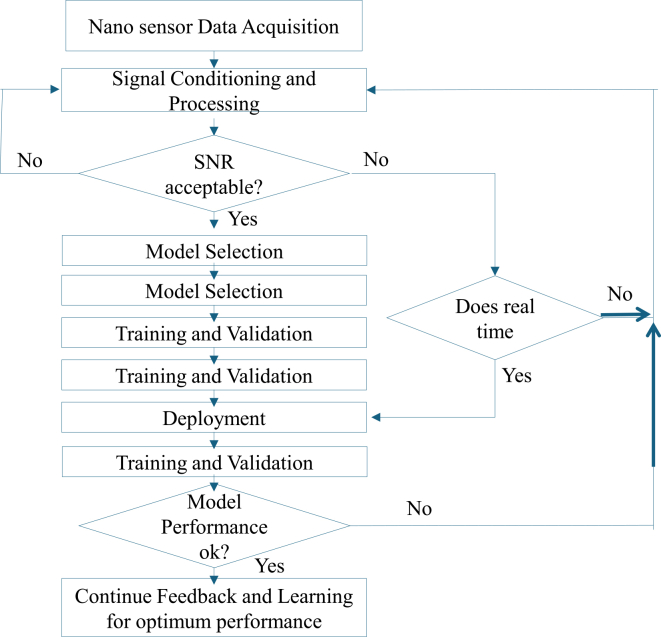
Workflow of ML Integration in Nanosensor Data Processing

Figure 5 shows the workflow of ML integration in nanosensor data processing, providing a comprehensive scientific architecture that maps the data journey from nanosensor signal acquisition to intelligent decision-making through machine learning.^143^^,^^151^ At its core, this framework reflects the hierarchical flow of nanoscale data through progressively complex computational stages, which transform raw sensor inputs into actionable insights.^144^^,^^145^^,^^152^^,^^153^ The architecture begins with the Nanosensor Data Acquisition Layer, where nanodevices collect environmental, physiological, or biochemical data.^154^^,^^155^ These sensors, due to their high sensitivity and low power consumption, are crucial in precision monitoring tasks such as biomedical diagnostics, environmental sensing, and smart agriculture.^156^ However, they often produce noisy, non-linear, and high-frequency signals that require conditioning.^157^ The preprocessing and signal conditioning stage addresses signal integrity challenges through amplification, filtering (Kalman filtering or bandpass filtering), and normalization techniques.^158^ This layer is vital to improving the SNR and preparing data for the downstream stages.^159^ A notable decision block at this point checks the SNR threshold, ensuring that only quality data proceed further, which prevents compounding errors in later ML stages.^160^ In high-dimensional sensing systems, this step may also include denoising algorithms and data interpolation to recover missing signals, especially in time-series biomedical or environmental applications where data continuity is essential.^161^ Subsequently, the Data Fusion and Feature Engineering Layer performs multi-sensor data integration and extraction of meaningful features. Data fusion techniques such as Bayesian inference or PCA are used to consolidate disparate sensor readings into a cohesive dataset, enabling holistic interpretation.^162^ Feature engineering then isolates the most informative variables using methods like mutual information or autoencoders.^163^ At this point, the system applies decision logic to validate the statistical relevance of extracted features.^146^ This is a critical aspect for reducing computational load in later stages and avoiding overfitting in ML models, particularly when deploying deep learning models on edge devices.^164^ In the Model Selection and Training Layer, the workflow adapts based on the nature and structure of the dataset whether labeled or unlabeled, static or streaming.^165^ Supervised learning models in SVM, RF are used for classification and regression tasks when labels are available, while unsupervised methods (clustering or dimensionality reduction) help in anomaly detection or pattern recognition when labels are absent.^166^ This layer also supports RL in dynamic or decision-critical environments such as autonomous sensing systems.^167^ The model training process includes hyperparameter optimization (grid search or Bayesian methods), cross-validation, and performance evaluation using metrics like accuracy, F1-score, or mean absolute error.^168^ Finally, the Deployment and Feedback Layer operationalizes the trained models across edge, fog, or cloud platforms depending on latency, power, and computational constraints.^169^ The workflow integrates lightweight AI models (TinyML, quantized neural nets) for edge deployment, while deep inference models are deployed in the cloud for large-scale analytics.^170^ Importantly, a feedback loop ensures real-time model revalidation and adaptation through techniques such as concept drift detection or online learning.^171^^,^^172^ This loop is essential for sustaining long-term model accuracy in non-stationary environments, making the entire system intelligent, adaptive, and resilient. Overall, this scientifically grounded workflow facilitates scalable, efficient, and intelligent processing of nanosensor data within modern ICT ecosystems.^147^^,^^173^^,^^174^^,^^175^^,^^176^ As nanosensor systems generate increasingly complex and high-volume data streams within ICT networks, the role of AI extends beyond signal interpretation to encompass data optimization and autonomous decision-making. By integrating AI at various layers of networked infrastructures, it becomes possible to enhance data flow efficiency, reduce latency, prioritize critical information, and enable intelligent resource allocation.^177^ The following section explores the transformative impact of AI-driven optimization and decision-making within networked ICT systems, highlighting its significance in achieving scalable, responsive, and context-aware nanosensor deployments.

## AI-driven data optimization and decision-making in networked ICT systems

**Figure 6:**
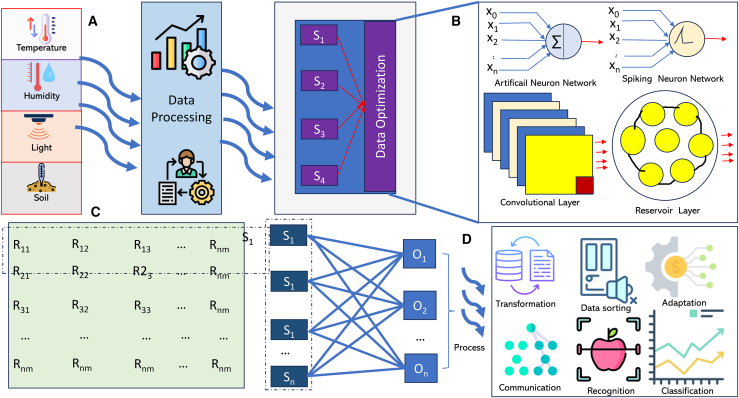
Integrated pipeline for an intelligent environmental monitoring system (A) transitioning from multi-modal sensor data acquisition. (B) through bio-inspired neural optimization. (C) hardware-efficient crossbar mapping. (D) real-time system applications.

Figure 6 presents a layered, modular architecture integrating nanosensors with AI-driven decision-making. This systematic layout spanning sensing, preprocessing, data optimization, AI modeling, and actuation aligns with traditional IoT architectures but introduces nanoscale sensing as the foundational innovation. Most prior works primarily describe macro-scale sensor networks with limited adaptability. The introduction of nanosensors in this figure reflects a shift toward ultra-precise, real-time contextual monitoring, a key demand in modern ICT applications.^148^^,^^178^ While traditional WSNs often utilize coarse-grained sensors (temperature, humidity), the nanosensor network here enables detection at molecular or atomic resolution. Compared with conceptual nanonetworks, the present architecture adds depth by coupling these sensors with immediate AI processing.^179^^,^^180^ This contrasts with older frameworks that required external servers for analysis, increasing latency and reducing responsiveness especially in time-sensitive domains like disease detection or smart agriculture, signal preprocessing, and edge intelligence.^181^^,^^182^ The inclusion of preprocessing at the sensor node level is a major advancement over legacy IoT models. Literature by Natarajan et al.^183^ on edge computing advocates pushing intelligence to the “edge” to reduce latency and bandwidth usage. The figure goes a step further by integrating this with nanosensors implying ultralightweight preprocessing techniques at the molecular level. However, the computational limits of nanoscale devices remain a concern, and future iterations may need hybrid approaches using microcontrollers or neuromorphic chips.^184^ Many IoT studies underemphasize the Human–Machine Interface (HMI), focusing instead on backend processing. Your figure places it prominently in architecture, suggesting a human-in-the-loop paradigm that increases transparency and trust. Unlike other AI systems criticized in some literature, this system could allow users to inspect raw data and model predictions. Sensor selection and data dimensionality reduction are actively researched topics. Most prior systems use manual feature selection or basic algorithms like PCA.^185^ In contrast, the current framework proposes an AI-optimized, adaptive sensor selection mechanism, where data streams are pruned dynamically based on real-time relevance.^186^ This innovation echoes the concept of self-optimizing sensor networks discussed by Nwabueze et al.,^187^ though this model extends it with real-time AI feedback loops for superior performance. The study further presents multi-algorithm and ensemble learning, a current best practice in AI. Many existing systems apply a single model type, resulting in sub-optimal outcomes across varied contexts. This framework suggests a modular approach where different models (SVM for classification, CNNs for vision tasks) are used in tandem or based on application-specific needs, aligning with the AutoML and transfer learning paradigms emerging in the edge-AI literature.^141^ In contrast to older sensor systems that only report data, this architecture feeds outputs into automated actuation mechanisms—enabling real-time control.^188^ Such closed-loop systems are rare in the nanosensor literature due to complexity and power constraints.^189^ However, this model shows clear paths from AI outputs to hardware action, reminiscent of cyber-physical systems but uniquely powered by nanoscale inputs for precision agriculture, environmental remediation, or biomedical implants.^190^ The application in speech recognition, computer vision (apple detection), and AI predictions demonstrates the system’s multimodal intelligence.^191^ Few nanosensor-integrated systems in the literature achieve this versatility.^192^ Some literature demonstrated deep learning in agriculture using only visual data, while this system incorporates voice interaction and predictive analytics, which could enhance usability for non-technical users and optimize deployment in rural or resource-limited settings.^193^ Contextual knowledge enables explainable and reasoned outputs from AI models, facilitating better decision-making.^194^ Your system appears to support ongoing learning and improved interpretability, addressing the growing demand of complex data processing.^195^

The integration of nanosensors, advanced communication technologies, and AI-driven analytics has laid a strong foundation for intelligent, responsive, and interconnected systems. However, several critical challenges and research opportunities remain. Advancements in energy-efficient designs, real-time learning at the edge, secure data exchange, and seamless interoperability will be pivotal for scaling these technologies. The following section outlines key future directions, offering insights into the emerging trends, technological gaps, and interdisciplinary innovations that will shape the next generation of nano-enabled ICT systems.^196^

## Dataset and research gap

Recent advancements in ML and IoT systems have led to the development of several domain-specific datasets such as the Intel Berkeley Research Lab dataset for environmental monitoring, the HealthIoT-ECG dataset for healthcare applications, and the IoT-23 dataset for cybersecurity analysis (Table 3).^199^^,^^200^ While these datasets have enabled targeted applications of supervised and unsupervised learning algorithms, including RF, SVM, Long Short-Term Memory (LSTM), and CNNs, they remain limited in terms of scalability, real-time adaptability, and nano-level sensing.^201^ For example, the UCI Air Quality dataset and the Intel Berkeley dataset offer useful temporal resolution, yet they lack nanoscale pollutant detection or the ability to interact with adaptive feedback systems. Likewise, biometric datasets like HealthIoT-ECG and Wearable Stress datasets focus on a single modality (ECG or stress) and do not incorporate the richness of biochemical data obtainable through nanosensors. A significant gap exists in the multimodal integration of datasets and the dynamic interoperability between ML models and heterogeneous sensor networks. Current condition monitoring datasets often lack domain-specific customization and are not scalable to diverse physical systems such as nano-enabled smart homes or precision agriculture platforms.^202^ The IoT-23 dataset, while offering useful insights into IoT traffic anomalies, is largely based on predefined attack scenarios, and it fails to capture emerging threat patterns relevant to nanoscale communication channels. Similarly, datasets used in smart infrastructure applications lack context-awareness and adaptability, limiting their relevance to real-time and unforeseen behaviors.^198^^,^^203^^,^^204^ These deficiencies restrict the generalizability of AI solutions and prevent meaningful deployment in complex, real-world environments. To address these critical gaps, our review highlights the necessity of a unified framework that integrates nanoscale sensors with ICT infrastructure and intelligent ML algorithms. By combining ultra-sensitive nanosensors for physiological, environmental, and biochemical detection with real-time data analytics and contextual learning models, we propose a paradigm shift in data collection, processing, and decision-making.^205^ This framework not only enables fine-grained monitoring and prediction but also supports adaptive behavior through continuous feedback loops and RL. Unlike traditional datasets that are fixed in structure and scope, the proposed system promotes a modular, self-calibrating architecture that evolves with user context and environmental change.^206^ In doing so, our review bridges fragmented research across multiple domains, offering a cohesive roadmap for future nano-IoT-ML systems. It contributes to the literature by providing an integrative perspective that emphasizes cross-domain interoperability, long-term scalability, multimodal sensor fusion, and real-time responsiveness. The proposed approach also lays the foundation for ethical and secure deployment of nano-enabled devices by incorporating privacy-preserving anomaly detection and edge AI models. Overall, this study positions itself at the intersection of next-generation computing, nanotechnology, and intelligent sensing offering a critical framework for advancing healthcare, environmental monitoring, infrastructure automation, and cyber-physical security. In summary, the datasets utilized across these studies span diverse domains including environmental monitoring, healthcare, IoT security, and precision medicine. The Intel Berkeley Research Lab and UCI Air Quality datasets focus on environmental sensing, featuring millions of time-series entries analyzed using supervised learning models such as SVM, RF, and KNN. In contrast, the Binary Data and IoT-23 datasets emphasize condition and network monitoring, leveraging unsupervised and anomaly detection techniques including autoencoders. Within healthcare and precision medicine, datasets like HealthIoT-ECG, Wearable Stress, and Wearable Sensor provide rich physiological and biometric signals—spanning thousands of patients or annotated windows—processed through advanced deep learning models such as CNNs, LSTMs, and RL. Additionally, the SmartHome Data capture over 100,000 activity logs with hybrid CNN-RNN architectures for intelligent infrastructure analysis. Collectively, these datasets show the versatility of AI algorithms in managing heterogeneous, large-scale IoT and biomedical data streams. Thus, existing open-access datasets across domains such as environmental monitoring, healthcare, IoT security, and precision medicine, such as the Intel Berkeley Research Lab, IoT-23, HealthIoT-ECG, SmartHome, and Wearable Sensor datasets, demonstrate the broad applicability of AI in managing large-scale time-series and physiological data. These datasets, ranging from millions of sensor readings to thousands of patient and activity records, have enabled advancements through algorithms like SVM, RF, CNN, LSTM, and Autoencoder. However, despite these resources, there remains a notable scarcity of nanosensor-specific datasets, particularly those capturing multimodal data integration across chemical, optical, and biological sensing domains. This limitation restricts the generalization and scalability of AI models in nanosensor-enabled ICT systems. Therefore, establishing standardized data collection protocols and open-access frameworks tailored for nanosensor research is essential to accelerate innovation and cross-domain model development.

**Table 3:** Summary of the dataset

Dataset Name	Type	Domain	Size	AI Algorithm Applied	Study
Intel Berkeley Research Lab	Time-series Sensor Data	Environmental Monitoring	2.3M rows, 54 nodes	Supervised Learning (SVM, RF)	Princz et al.^22^
Binary data	Time-series Sensor Data	Condition Monitoring	–	Unsupervised Machine learning	Ahmed et al.^1^
HealthIoT-ECG	Biometric Signals (ECG)	Healthcare	12,000+ patients	Deep CNN, LSTM	Wang et al.^197^
IoT-23	Network Traffic Logs	IoT Security	1.1M flows, 20 scenarios	Anomaly Detection (Autoencoder)	Xiong et al.^98^
UCI Air Quality Dataset	Environmental	Air Pollution Monitoring	9,358 hourly entries	Random Forest, KNN	–
SmartHome Data	Binary/Multiclass Labels	Smart Infrastructure	100,000+ activity logs	Hybrid CNN-RNN	Azab et al.^198^
Wearable Stress Dataset	Physiological Signals	Precision Medicine	6,000+ annotated windows	SVM, LSTM, Reinforcement Learning	Liu et al.^115^
Wearable Sensor Dataset	Physiological Signals	Precision Medicine	6,000+ annotated windows	SVM, LSTM, Reinforcement Learning	Shetty et al.^28^

The integration of nanosensor networks with advanced communication protocols such as LoRaWAN, Zigbee, and 6LoWPAN enables long-range, low-power data transmission, positioning them as key enablers for precision agriculture, industrial safety, and real-time environmental security systems. These protocols provide scalable and energy-efficient data routing mechanisms critical for nanoscale devices that often operate under constrained power and bandwidth conditions. Nonetheless, persistent challenges such as electromagnetic interference, limited ruggedization for harsh environments, and degradation in long-term sensor reliability impede practical deployment. Furthermore, the lack of standardized datasets for nanosensor network performance evaluation limits the benchmarking and reproducibility of AI/ML algorithms designed for such systems. To bridge this gap, several IoT datasets have been explored to emulate nanosensor environments and support AI-driven modeling. Table 3 summarizes representative datasets frequently employed in nanosensor-related IoT studies. These include the Intel Berkeley Research Lab dataset—time-series environmental sensor data comprising 2.3 million readings across 54 nodes, extensively used for supervised learning and regression analyses; HealthIoT-ECG, which contains over 12,000 patient records for biomedical signal processing via deep convolutional and recurrent neural architectures; and IoT-23, a large-scale IoT network traffic dataset (1.1M flows across 20 scenarios) used for cybersecurity anomaly detection employing autoencoder-based frameworks. Similarly, the UCI Air Quality Dataset offers environmental data used for air pollution modeling using RF and KNN algorithms, while SmartHome Data support hybrid CNN-RNN learning for smart infrastructure activity recognition. Physiological datasets such as the Wearable Stress Dataset and Wearable Sensor Dataset are also utilized in precision medicine for stress and activity detection using SVM, LSTM, and RL approaches.^28^ Despite their relevance, these datasets primarily capture macroscale or IoT-level phenomena, underscoring the urgent need for nanosensor-specific datasets that account for unique operational constraints, such as energy budgets, molecular-scale noise, and limited transmission capacity. Developing such datasets remains a key prerequisite for advancing explainable and data-efficient AI models tailored for nanosensor-based ICT systems.^207^

The comparative analysis in Table 4 highlights the distinct contributions of the present review in relation to the existing literature. Prior studies, such as those focusing on AI for IoT sensor networks and general ICT frameworks, primarily addressed macro-scale systems with limited applicability to nanoscale communication environments. Although these works explored optimization and routing mechanisms, they lacked attention to nanosensor constraints, including limited energy budgets, high noise levels, and privacy-sensitive data exchange. Similarly, reviews centered on nanosensor communication provided valuable insights into physical and MAC-layer design but did not explore the potential of AI or ML integration for intelligent adaptation and system-level optimization. ML-focused reviews advanced algorithmic discussions but often neglected the complexities introduced by nanoscale device heterogeneity and dynamic environmental interactions. In contrast, the present study bridges these gaps by synthesizing AI-driven routing techniques, edge-cloud collaboration models, and layered ICT architectures explicitly tailored for nanosensor networked systems. Moreover, this work uniquely integrates technical and non-technical dimensions including regulatory, ethical, and data security challenges providing a holistic framework for future development. Overall, this critical comparison underscores that, while existing reviews contribute valuable domain-specific insights, the current study advances the field by presenting a unified, interdisciplinary perspective that connects nanosensor technology, AI/ML models, and intelligent ICT infrastructures for next-generation, adaptive, and secure nanosensor networks.^208^^,^^209^^,^^210^^,^^211^^,^^212^^,^^213^

**Table 4:** Comparison of existing reviews and the present study on AI-driven routing and layered architectures for intelligent ICT in nanosensor networked systems

Study/Review	Main Focus	Scope & Coverage	Limitations of Existing Reviews	Key Contributions of the Present Work
AI for IoT Sensor Networks	Focuses on AI-based optimization and routing in general IoT systems	Discusses classical ML techniques and network efficiency	Limited attention to nanoscale communication and sensing layers	Integrates nanosensor-based ICT frameworks with AI-driven routing and layered architectures
Nanosensor Communication and Networking	Examines nanonetwork protocols and THz communication models	Strong focus on physical and MAC layers	Lacks discussion on AI/ML integration and adaptive decision systems	Introduces AI-enhanced layered architecture linking nanosensor data processing to ICT layers
Machine Learning in Sensor Networks	Reviews supervised and unsupervised ML for WSNs	Addresses data analytics and model selection	No emphasis on nanoscale device limitations or energy-efficient routing	Provides ML-driven optimization for nanosensor routing and data fusion
ICT Frameworks for Smart Systems	Focuses on general ICT infrastructures	Covers cloud-edge computing and data management	Limited to macro-scale systems; minimal nanosensor consideration	Extends ICT frameworks to nanosensor systems emphasizing edge intelligence and federated learning
Present Review	AI-Driven Routing and Layered Architectures for Intelligent ICT in Nanosensor Networked Systems	Integrates nanosensor communication, AI/ML algorithms, and ICT infrastructures for intelligent decision-making and system optimization	Bridges the gap between nanosensor technology and AI-enabled ICT frameworks, addressing both technical and non-technical challenges (privacy, regulation, and scalability)	Linked nanosensor technology and AI-enabled ICT frameworks for optimum operation

## Future research directions

Despite significant advancements, several critical research frontiers remain open for the development of intelligent, scalable nanosensor networked systems. The next generation of nano-ICT infrastructures must focus on designing scalable, energy-efficient AI algorithms that are specifically tailored to the physical and power constraints of nanoscale devices. Traditional deep learning architectures are computationally intensive and unsuitable for direct deployment on nanosensors. Thus, future work should prioritize lightweight, resource-constrained AI models such as binary neural networks, quantized learning approaches, and spiking neural networks that enable decentralized inference with minimal hardware overhead. Progress in neuromorphic computing and on-chip AI integration at the nanoscale will be pivotal in this domain. A foundational research challenge lies in the formal mathematical modeling and verification of AI-driven communication protocols in nanosensor networks. As these networks become more complex and dynamic, there is an urgent need for analytical models that can predict system behavior, optimize communication routes, and ensure network stability and security. Future research should explore the application of game theory, Markov decision processes, and control theory to mathematically establish performance guarantees, convergence properties, and resilience under dynamic and adversarial conditions. Another promising avenue is the integration of FL and swarm intelligence paradigms within nanosensor environments. FL allows distributed AI training while preserving data privacy—crucial for applications in biomedicine and defense. Swarm intelligence methods (e.g., ant colony optimization, particle swarm optimization) offer biologically inspired mechanisms for distributed decision-making, clustering, and adaptive routing in large-scale nano-IoT systems. Coupling these paradigms with graph-based mathematical models may enable hierarchical coordination and intelligent load balancing across the network. Looking further ahead, the fusion of quantum communication and AI-enabled nanosensor systems represents a transformative research direction. With emerging technologies such as quantum-dot sensors and quantum key distribution, there is a need to develop quantum-enhanced ML frameworks capable of facilitating secure, ultra-low-latency communication at the nanoscale. Formal abstraction of quantum-AI interaction models will be necessary to design robust hybrid architectures that integrate classical and quantum communication protocols. Finally, addressing the lack of standardized simulation environments and benchmarking tools is essential for bridging the gap between theory and practice. Current models often fail to capture the physical and communication constraints inherent to nanosensor networks. Future work must prioritize the development of AI-integrated simulators, standardized testbeds, and open-access datasets that reflect realistic operating conditions. These tools will be instrumental in enabling reproducibility, comparative performance evaluation, and real-world deployment. Furthermore, advancing this field will require interdisciplinary collaboration among material scientists, AI researchers, and communication engineers to co-design integrated hardware-software systems that realize the full potential of intelligent nano-ICT infrastructures. Future nanosensor dataset creation should follow rigorous scientific and technical standards to ensure reproducibility, scalability, and AI-readiness. Datasets must be context-specific, reflecting the sensing objectives and operational environments of nanosensor systems in biomedical, environmental, or industrial domains. Multimodal data acquisition—integrating optical, electrochemical, and piezoresistive modalities—should be prioritized to enable robust feature fusion and cross-domain learning. Standardized metadata and labeling protocols are essential for interoperability and benchmarking, while noise-aware data collection must address nanoscale signal drift and cross-sensitivity through real-time filtering and adaptive calibration. When experimental data are limited, synthetic data generation using physics-informed models and transfer learning from large-scale IoT or biomedical datasets can enhance dataset diversity and generalization. Moreover, the establishment of collaborative open-access repositories adhering to FAIR (Findable, Accessible, Interoperable, Reusable) principles will promote transparency and accelerate innovation in nanosensor-AI research.

## Conclusion

The convergence of ML and AI with nanosensor-enabled ICT systems signifies a pivotal shift in the development of intelligent, adaptive, and scalable communication infrastructures. This review has systematically analyzed recent advancements in AI-driven architectures and routing strategies, addressing key challenges such as energy efficiency, latency reduction, scalability, and dynamic network topologies inherent to nanosensor networks. By integrating advanced mathematical models and optimization techniques—including graph-theoretic frameworks, stochastic processes, and deep learning methodologies—researchers have enhanced the robustness and adaptability of nanoscale communication protocols. AI-powered solutions, particularly those based on RL, FL, and neural network-based optimizers, have demonstrated significant potential in enabling self-organizing network behavior, real-time data analytics, and autonomous decision-making. These intelligent models, when embedded into multi-layered ICT frameworks, facilitate cross-layer coordination, secure data aggregation, and efficient resource management, even under the strict computational and environmental constraints of nanoscale systems. Moreover, a growing emphasis on hybrid approaches—combining model-based inference with data-driven learning—underscores the importance of precision and generalization in achieving practical, real-world deployment across applications such as biomedical diagnostics, environmental monitoring, and nano-cyberphysical systems. Looking forward, the continued evolution of this field will depend on the development of formally verified AI protocols, secure and energy-efficient multi-agent learning frameworks, and real-time optimization algorithms tailored to the constraints of nanoelectronic hardware. The integration of ML and AI into the mathematical and architectural foundations of nanosensor-based ICT systems not only enhances operational performance but also lays a scalable and intelligent groundwork for the future of autonomous, adaptive, and resilient nano-enabled communication networks. The novelty of this review lies in its technical synthesis of nanosensor networks with advanced ICT and AI frameworks, offering a unified analysis of learning algorithms, edge_cloud_federated architectures, and AI-optimized communication protocols. It critically evaluates system performance in terms of latency, energy efficiency, and inference accuracy; identifies key challenges in scalability, interoperability, and data security; and introduces emerging paradigms such as bio-inspired, explainable, and quantum-enhanced learning for developing intelligent and adaptive nanosensor_ICT systems.

## Acknowledgments

This research has been funded by 10.13039/501100023674Scientific Research Deanship at 10.13039/501100008809University of Hail - Saudi Arabia through project RG-24 186.

## Author contributions

A.K.Y.D. contributed to the identification and evaluation of relevant sources, data collection, and drafting of the manuscript. T.A.A.G. participated in the review of relevant sources and manuscript writing. A.F. contributed to data analysis and critical revision of the manuscript. N.M.O.S.A. assisted in data interpretation and manuscript preparation. T.A. conceptualized and supervised the study and critically revised the manuscript. M.E.E. contributed to the study design and reviewed the manuscript. S.C.B.G. contributed to critical revision and final approval of the manuscript.

## Declaration of interests

The authors declare no competing interests.

## Declaration of generative AI and AI-assisted technologies in the writing process

During the preparation of this work, the authors did not use any AI-assisted tools or services. The authors take full responsibility for the content of the publication.

## References

[1] Ahmed B.T., Mustafa Abdulrahm Z., Addie A.J., Haider A.J., Alkawaz A.N., Yaqoob I.A.M., Arsad N. (2025). Advancing optical nanosensors with artificial intelligence: A powerful tool to identify disease-specific biomarkers in multi-omics profiling. Talanta.

[2] Galal A., Hesselbach X. (2018). Nano-networks communication architecture: Modeling and functions. Nano Commun. Netw..

[3] Tang H., Kong L., Fang Z., Zhang Z., Zhou J., Chen H., Sun J., Zou X. (2024). Sustainable and smart rail transit based on advanced self-powered sensing technology. iScience.

[4] Olawade D.B., Ige A.O., Olaremu A.G., Ijiwade J.O., Adeola A.O. (2024). The synergy of artificial intelligence and nanotechnology towards advancing innovation and sustainability - A mini-review. Nano Trends.

[5] Mohsan S.A.H., Li Y. (2023). A Contemporary Survey on 6G Wireless Networks: Potentials, Recent Advances, Technical Challenges and Future Trends. arXiv.

[6] Balghusoon A.O., Mahfoudh S. (2020). Routing Protocols for Wireless Nanosensor Networks and Internet of Nano Things: A Comprehensive. IEEE Access.

[7] Khan L.U., Yaqoob I., Imran M., Han Z., Hong C.S. (2020). 6G Wireless Systems: A Vision, Architectural Elements, and Future Directions. IEEE Access.

[8] Oukhatar A., Bakhouya M., Ouadghiri D.E. (2021). Electromagnetic-based wireless nano-sensors network: Architectures and applications. J. Commun..

[9] Yu W., Liang F., He X., Hatcher W.G., Lu C., Lin J., Yang X. (2018). A Survey on the Edge Computing for the Internet of Things. IEEE Access.

[10] Arellano Vidal C.L., Govan J.E. (2024). Machine Learning Techniques for Improving Nanosensors in Agroenvironmental Applications. Agronomy.

[11] Arellano Vidal C.L., Govan J.E. (2024). Machine Learning Techniques for Improving Nanosensors in Agroenvironmental Applications. Agronomy.

[12] Veeralingam S., Khandelwal S., Badhulika S. (2020). AI/ML-Enabled 2-D - RuS2Nanomaterial-Based Multifunctional, Low Cost, Wearable Sensor Platform for Non-Invasive Point of Care Diagnostics. IEEE Sens. J..

[13] Ang L.M., Seng K.P., Wachowicz M. (2022). Embedded intelligence and the data-driven future of application-specific Internet of Things for smart environments. Int. J. Distrib. Sens. Netw..

[14] Leong S.X., Leong Y.X., Koh C.S.L., Tan E.X., Nguyen L.B.T., Chen J.R.T., Chong C., Pang D.W.C., Sim H.Y.F., Liang X. (2022). Emerging nanosensor platforms and machine learning strategies toward rapid, point-of-need small-molecule metabolite detection and monitoring. Chem. Sci..

[15] Garcia-Sanchez A.J., Asorey-Cacheda R., Garcia-Haro J., Gomez-Tornero J.L. (2023). Dynamic Multihop Routing in Terahertz Flow-Guided Nanosensor Networks: A Reinforcement Learning Approach. IEEE Sens. J..

[16] Yang L., Wang H., Leng D., Fang S., Yang Y., Du Y. (2024). Machine learning applications in nanomaterials: Recent advances and future perspectives. Chem. Eng. J..

[17] Musa S.S., Ibrahim A.M., Alhassan M.Y., Musa A.H., Jibo A.G., Auwal A.R., Okesanya O.J., Othman Z.K., Abubakar M.S., Ahmed M.M. (2025). Nanotechnology and machine learning: a promising confluence for the advancement of precision medicine. Intell. Based Med..

[18] Ray S. (2019). Proc. 2019 Int. Conf. Machine Learning, Big Data, Cloud and Parallel Computing (COMITCon).

[19] Winkler D.A. (2020). Role of Artificial Intelligence and Machine Learning in Nanosafety. Small.

[20] Nandipati M., Fatoki O., Desai S. (2024). Bridging Nanomanufacturing and Artificial Intelligence A Comprehensive Review. Materials.

[21] Hassan S.A.H., Almaliki M.N.S., Hussein Z.A., Albehadili H.M., Rabeea Banoon S., Abboodi A., Al-Saady M. (2023). Development of Nanotechnology by Artificial Intelligence: A Comprehensive Review. J. Nanostructures.

[22] Princz G., Shaloo M., Erol S. (2024). Anomaly Detection in Binary Time Series Data: An unsupervised Machine Learning Approach for Condition Monitoring. Procedia Comput. Sci..

[23] Tahir N., Parasuraman R. (2025). Edge Computing and Its Application in Robotics: A Survey. J. Sens. Actuator Netw..

[24] Wu N., Sun Y., Hu J., Yang C., Bai Z., Wang F., Cui X., He S., Li Y., Zhang C. (2025). Intelligent nanophotonics: when machine learning sheds light. eLight.

[25] Agboklu M., A Adrah F., Agbenyo P.M., Nyavor H. (2024). From Bits to Atoms: Machine Learning and Nanotechnology for Cancer Therapy. J. Nanotechnol. Res..

[26] Wang X., Jia W. (2025). Optimizing Edge AI: A Comprehensive Survey on Data, Model, and System Strategies. arXiv.

[27] Kalra P. (2022). Mathematical models for Machine Learning Techniques. J. Emerg. Technol. Innov. Res..

[28] Shetty C., Prasad P. (2025). AI-Optimized Design of Nanosensors for Real-Time Pathogen Detection. IJSRED.

[29] Pancino N., Perron Y., Bongini P., Scarselli F. (2022). Drug Side Effect Prediction with Deep Learning Molecular Embedding in a Graph-of-Graphs Domain. Mathematics.

[30] Sulaiman M., Khalaf O.I., Khan N.A., Alshammari F.S., Hamam H. (2024). Mathematical modeling and machine learning-based optimization for enhancing biofiltration efficiency of volatile organic compounds. Sci. Rep..

[31] Kuo C.N., Cheng Y.H. (2025). Fault-Tolerant Path Embedding in Folded Hypercubes Under Conditional Vertex Constraints. Mathematics.

[32] Chen S., Wu G. (2025). Memory-Based Differential Evolution Algorithms with Self-Adaptive Parameters for Optimization Problems. Mathematics.

[33] Yang X., Yang L. (2025). Numerical Simulation of High-Pressure Water Jets in Air by an Elliptic–Blending Turbulence Model: A Parametric Study. Mathematics.

[34] Muthuvel P., Gopal P., Manickam S., Chellappan R. (2024). Optimizing Road Networks: A Graph-Based Analysis with Path-finding and Learning Algorithms. Int. J. ITS. Res..

[35] Yan Y. (2023). Research on the A Star Algorithm for Finding Shortest Path. Highlights Sci. Eng. Technol..

[36] Zhang Z., Liu X., Zhou H., Xu S., Lee C. (2024). Advances in Machine-Learning Enhanced Nanosensors: From Cloud Artificial Intelligence Toward Future Edge Computing at Chip Level. Small Struct..

[37] Soori M., Arezoo B., Dastres R. (2023). Artificial intelligence, machine learning and deep learning in advanced robotics, a review. Cognit. Robot..

[38] Ugwoke K.C., Nnanna N.A., Abdullahi S.E.Y. (2025). Simulation-based review of classical, heuristic, and metaheuristic path planning algorithms. Sci. Rep..

[39] Harrou F., Zeroual A., Kadri F., Sun Y. (2024). Enhancing road traffic flow prediction with improved deep learning using wavelet transforms. Results Eng..

[40] Antikainen H. (2013). Using the Hierarchical Pathfinding A∗ Algorithm in GIS to Find Paths through Rasters with Nonuniform Traversal Cost. ISPRS Int. J. GeoInf..

[41] Collins E., Wang M. (2025). Federated Learning: A Survey on Privacy-Preserving Collaborative Intelligence. arXiv.

[42] Orabi M.M., Emam O., Fahmy H. (2025). Adapting security and decentralized knowledge enhancement in federated learning using blockchain technology: literature review. J. Big Data.

[43] Lazaros K., Koumadorakis D.E., Vrahatis A.G., Kotsiantis S. (2024). Federated Learning: Navigating the Landscape of Collaborative Intelligence. Electronics (Switzerland).

[44] Li T., Sahu A.K., Talwalkar A., Smith V. (2020). Federated Learning: Challenges, Methods, and Future Directions. IEEE Signal Process. Mag..

[45] Tanoli S.A.K., Rehman M., Khan M.B., Jadoon I., Ali Khan F., Nawaz F., Shah S.A., Yang X., Nasir A.A. (2018). An Experimental Channel Capacity Analysis of Cooperative Networks Using Universal Software Radio Peripheral (USRP). Sustainability.

[46] Kumar K.A.S., Nelson L., Jibinsingh B.R. (2025). Systematic review of privacy-preserving Federated Learning in decentralized healthcare systems. Franklin Open.

[47] Ziółkowski C., Kelner J.M., Krygier J., Chandra A., Prokeš A. (2021). Radio channel capacity with directivity control of antenna beams in multipath propagation environment. Sensors.

[48] Bi S., Zeng Y., Zhang R. (2016). Wireless powered communication networks: An overview. IEEE Wirel. Commun..

[49] Cai N., Yeung R.W. (2011). Secure Network Coding on a Wiretap Network. Information Theory. IEEE Transactions on.

[50] Butzer P.L., Schmeisser G., Stens R.L. (2012). Shannon’s sampling theorem for bandlimited signals and their hilbert transform, boas-type formulae for higher order derivatives-the aliasing error involved by their extensions from bandlimited to non-bandlimited signals. Entropy.

[51] Koyuncu E. (2024). Information Theory in Emerging Wireless Communication Systems and Networks. Entropy.

[52] Santoso B., Oohama Y. (2019). Information theoretic security for Shannon cipher system under side-channel attacks. Entropy.

[53] Sarker I.H. (2021). Machine Learning: Algorithms, Real-World Applications and Research Directions. SN Comput. Sci..

[54] Paunovska K., Loughrey D., Sago C.D., Langer R., Dahlman J.E. (2019). Using Large Datasets to Understand Nanotechnology. Adv. Mater..

[55] Rana A., Gautam D., Kumar P., Kumar K., Vasilakos A.V., Das A.K., K V.B. (2025). A comprehensive review of machine learning applications for internet of nano things: challenges and future directions. Artif. Intell. Rev..

[56] Taha T.B., Barzinjy A.A., Hussain F.H.S., Nurtayeva T. (2022). Nanotechnology and Computer Science: Trends and advances. Memories - Materials, Devices, Circuits and Systems.

[57] Alzubi J., Nayyar A., Kumar A. (2018). Machine Learning from Theory to Algorithms: An Overview. J. Phys, Conf. Ser..

[58] Adir O., Poley M., Chen G. (2021). Europe PMC Funders Group Integrating Artificial Intelligence and Nanotechnology for Precision. Cancer Med..

[59] Song L., Hu X., Zhang G., Spachos P., Plataniotis K.N., Wu H. (2022). Networking Systems of AI: On the Convergence of Computing and Communications. IEEE Internet Things J..

[60] Masson J.F. (2024). Roadmap for the Use of Machine Learning and Artificial Intelligence in Sensing. ACS Sens..

[61] Smith C.W., Hizir M.S., Nandu N., Yigit M.V. (2022). Algorithmically Guided Optical Nanosensor Selector (AGONS): Guiding Data Acquisition, Processing, and Discrimination for Biological Sampling. Anal. Chem..

[62] Revignas D., Amendola V. (2022). Artificial Neural Networks Applied to Colorimetric Nanosensors: An Undergraduate Experience Tailorable from Gold Nanoparticles Synthesis to Optical Spectroscopy and Machine Learning. J. Chem. Educ..

[63] Sun T., Feng B., Huo J., Xiao Y., Wang W., Peng J., Li Z., Du C., Wang W., Zou G., Liu L. (2023). Artificial Intelligence Meets Flexible Sensors: Emerging Smart Flexible Sensing Systems Driven by Machine Learning and Artificial Synapses. Nano-Micro Lett..

[64] Jafrasteh F., Farmani A., Mohamadi J. (2023). Meticulous research for design of plasmonics sensors for cancer detection and food contaminants analysis via machine learning and artificial intelligence. Sci. Rep..

[65] de Andrade L.P., Zorzo S.D., Santana M., Montealegre R. (2019). Proc. 25th Americas Conf. Information Systems, AMCIS 2019.

[66] Shajari S., Kuruvinashetti K., Komeili A., Sundararaj U. (2023). The Emergence of AI-Based Wearable Sensors for Digital Health Technology: A Review. Sensors.

[67] Zhang Z., Liu X., Zhou H., Xu S., Lee C. (2023). Advances in Machine-Learning Enhanced Nanosensors: From Cloud Artificial Intelligence Toward Future Edge Computing at Chip Level. Small Struct..

[68] Park S., Lee S., Park S., Park S. (2019). AI-based physical and virtual platform with 5-layered architecture for sustainable smart energy city development. Sustainability.

[69] Ospina Cifuentes B.J., Suárez Á., García Pineda V., Alvarado Jaimes R., Montoya Benitez A.O., Grajales Bustamante J.D. (2024). Analysis of the Use of Artificial Intelligence in Software-Defined Intelligent Networks: A Survey. Technologies.

[70] Wu J., Mo Z., Gao X., Xin W., Shi W., Park J. (2025). Artificial intelligence assisted wearable flexible sensors for sports: research progress in technology integration and application. Int. J. Smart Nano Mater..

[71] Kumar V., Sharma K.V., Kedam N., Patel A., Kate T.R., Rathnayake U. (2024). A comprehensive review on smart and sustainable agriculture using IoT technologies. Smart Agric. Technol..

[72] Soussi A., Zero E., Sacile R., Trinchero D., Fossa M. (2024). Smart Sensors and Smart Data for Precision Agriculture: A Review. Sensors.

[73] Hosny K.M., El-Hady W.M., Samy F.M. (2025). Technologies, Protocols, and applications of Internet of Things in greenhouse Farming: A survey of recent advances. Inf. Process. Agric..

[74] Aldhaheri L., Manzil I.I.J., Khalil R.A., Javaid S., Saeed N. (2025). LoRa Communication for Agriculture 4.0: Opportunities, Challenges, and Future Directions. IEEE Internet Things J..

[75] Mgendi G. (2024). Unlocking the potential of precision agriculture for sustainable farming. Discov. Agric..

[76] Dhakshayani J., Surendiran B., Jyothsna J. (2023). Artificial Intelligence in Precision Agriculture. Predictive Analytics in Smart Agriculture.

[77] Addas A., Tahir M., Ismat N. (2023). Enhancing Precision of Crop Farming towards Smart Cities: An Application of Artificial Intelligence. Sustainability.

[78] Getahun S., Kefale H., Gelaye Y. (2024). Application of Precision Agriculture Technologies for Sustainable Crop Production and Environmental Sustainability: A Systematic Review. Scientific World J..

[79] Yinjun Z. (2024). An adaptive hexagonal deployment model for resilient wireless sensor networks in precision agriculture. Sci. Rep..

[80] Bayih A.Z., Morales J., Assabie Y., de By R.A. (2022). Utilization of Internet of Things and Wireless Sensor Networks for Sustainable Smallholder Agriculture. Sensors.

[81] Chilamkurthy N.S., Pandey O.J., Ghosh A., Cenkeramaddi L.R., Dai H.N. (2022). Low-Power Wide-Area Networks: A Broad Overview of Its Different Aspects. IEEE Access.

[82] Kim C., Kang M.S., Raja I.S., Oh J.W., Joung Y.K., Han D.W. (2024). Current issues and perspectives in nanosensors-based artificial olfactory systems for breath diagnostics and environmental exposure monitoring. TrAC, Trends Anal. Chem..

[83] Thakur M., Wang B., Verma M.L. (2022). Development and applications of nanobiosensors for sustainable agricultural and food industries: Recent developments, challenges and perspectives. Environ. Technol. Innov..

[84] Malik S., Muhammad K., Waheed Y. (2023). Nanotechnology: A Revolution in Modern Industry. Molecules.

[85] Akhtar N., Perwej Y. (2020). The internet of nano things (IoNT) existing state and future Prospects. GSC Adv. Res. Rev..

[86] Zong B., Wu S., Yang Y., Li Q., Tao T., Mao S. (2024). Smart Gas Sensors: Recent Developments and Future Prospective. Nano-Micro Lett..

[87] Civas M., Kuscu M., Cetinkaya O., Ortlek B.E., Akan O.B., Akan O.B. (2023). Graphene and related materials for the Internet of Bio-Nano Things. APL Mater..

[88] Mazzetto S. (2024). A Review of Urban Digital Twins Integration, Challenges, and Future Directions in Smart City Development. Sustainability.

[89] Chaturvedi A., Tripathi D., Ranjan R. (2025). Nano-enabled biosensors in early detection of plant diseases. Front. Nanotechnol..

[90] Tregubov A.A., Nikitin P.I., Nikitin M.P. (2018). Advanced Smart Nanomaterials with Integrated Logic-Gating and Biocomputing: Dawn of Theranostic Nanorobots. Chem. Rev..

[91] Arroyo-Ortega I., Chavarin-Pineda Y., Torres E. (2024). Assessing Contamination in Transitional Waters Using Geospatial Technologies: A Review. ISPRS Int. J. GeoInf..

[92] Sharma P., Pandey V., Sharma M.M.M., Patra A., Singh B., Mehta S., Husen A. (2021). A Review on Biosensors and Nanosensors Application in Agroecosystems. Nanoscale Res. Lett..

[93] Boruah P.K., Sharma B., Hussain N., Das M.R. (2017). Magnetically recoverable Fe3O4/graphene nanocomposite towards efficient removal of triazine pesticides from aqueous solution: Investigation of the adsorption phenomenon and specific ion effect. Chemosphere.

[94] Islam S., Samsuzzaman, Reza M.N., Lee K.H., Ahmed S., Cho Y.J., Noh D.H., Chung S.O. (2024). Image Processing and Support Vector Machine (SVM) for Classifying Environmental Stress Symptoms of Pepper Seedlings Grown in a Plant Factory. Agronomy.

[95] Huang X., Zhu Y., Kianfar E. (2021). Nano Biosensors: Properties, applications and electrochemical techniques. J. Mater. Res. Technol..

[96] Zhu B., Ren G., Tang M., Chai F., Qu F., Wang C., Su Z. (2018). Fluorescent silicon nanoparticles for sensing Hg2+ and Ag+ as well visualization of latent fingerprints. Dyes Pigments.

[97] Li Y., Zhang W., Cui Z., Shi L., Shang Y., Ji Y., Wang J. (2024). Machine learning-assisted nanosensor arrays: An efficiently high-throughput food detection analysis. Trends Food Sci. Technol..

[98] Xiong L., Liu Y., Jiao Y., Wei G., Xu B., Han F., Zhao L. (2025). Spectrochimica Acta Part A : Molecular and Biomolecular Spectroscopy Fluorescence detection of α -amylase based on a host – guest complex between a pyrene-derived amphiphile and γ -cyclodextrin. Spectrochim. Acta Mol. Biomol. Spectrosc..

[99] Liu W., Dong J., Ren Y., Zhou W., Han Q., Zhang C., Ren K., Wang Y., Gao W., Qi J. (2025). Fabrication of plasmonic Au-Ag alloy nanostars for ultrasensitive SERS detection. Spectrochim. Acta. A Mol. Biomol. Spectrosc..

[100] Hemdan M., Abuelhaded K., Shaker A.A., Ashour M.M., Abdelaziz M.M., Dahab M.I., Nassar Y.A., Sarguos A.M., Zakaria P.S., Fahmy H.A. (2025). Recent advances in nano-enhanced biosensors: Innovations in design, applications in healthcare, environmental monitoring, and food safety, and emerging research challenges. Sens. Biosensing Res..

[101] Mim J.J., Hasan M., Chowdhury M.S., Ghosh J., Mobarak M.H., Khanom F., Hossain N. (2024). A comprehensive review on the biomedical frontiers of nanowire applications. Heliyon.

[102] Jalalvand A.R., Karami M.M. (2025). Roles of nanotechnology in electrochemical sensors for medical diagnostic purposes: A review. Sens. Biosensing Res..

[103] Darwish M.A., Abd-Elaziem W., Elsheikh A., Zayed A.A. (2024). Advancements in nanomaterials for nanosensors: a comprehensive review. Nanoscale Adv..

[104] Jang H., Lee W., Lee J. (2018). Nanoparticle dispersion with surface-modified silica nanoparticles and its effect on the wettability alteration of carbonate rocks. Colloids Surf. A Physicochem. Eng. Asp..

[105] Raghu M.S., Yogesh Kumar K., Prashanth M.K., Prasanna B.P., Vinuth R., Pradeep Kumar C.B. (2017). Adsorption and antimicrobial studies of chemically bonded magnetic graphene oxide-Fe3O4 nanocomposite for water purification. J. Water Process Eng..

[106] Rawtani D., Khatri N., Tyagi S., Pandey G. (2018). Nanotechnology-based recent approaches for sensing and remediation of pesticides. J. Environ. Manage..

[107] Ghosh T., Raj G.V.S.B., Dash K.K. (2022). A comprehensive review on nanotechnology based sensors for monitoring quality and shelf life of food products. Measurement: Food.

[108] Ghorbian M., Ghobaei-Arani M., Babaei M.R., Ghorbian S. (2025). Nanotechnology and nanosensors in personalized healthcare: A comprehensive review. Sens. Biosensing Res..

[109] Sharma M., Mahajan P., Alsubaie A.S., Khanna V., Chahal S., Thakur A., Yadav A., Arya A., Singh A., Singh G. (2025). Next-generation nanomaterials-based biosensors: Real-time biosensing devices for detecting emerging environmental pollutants. Mater. Today Sustain..

[110] Zhou S., Chen R., Hou Y., Zou L., Wang R., Li G. (2025). Nanozyme-Facilitated In-Situ Fluorogenic Ratiometric Nanosensor for Ultrasensitive and Selective Dopamine Detection. J. Fluoresc..

[111] Kulkarni M.B., Ayachit N.H., Aminabhavi T.M. (2022). Recent Advancements in Nanobiosensors: Current Trends, Challenges, Applications, and Future Scope. Biosensors.

[112] Islam S., Shukla S., Bajpai V.K., Han Y.K., Huh Y.S., Kumar A., Ghosh A., Gandhi S. (2019). A smart nanosensor for the detection of human immunodeficiency virus and associated cardiovascular and arthritis diseases using functionalized graphene-based transistors. Biosens. Bioelectron..

[113] Saylan Y., Denizli A., Han B., Tomer V.K., Nguyen T.A., Farmani A., Singh P.K. (2020). Nanosensors for Smart Cities.

[114] Javaid M., Haleem A., Singh R.P., Rab S., Suman R. (2021). Exploring the potential of nanosensors: A brief overview. Sens. Int..

[115] Liu R., Song Z., Li Y., Li Y., Yao W., Sun H., Zhu H. (2018). An AIPE-active heteroleptic Ir(III) complex for latent fingermarks detection. Sens. Actuators, B.

[116] Zhang H., You J., Nie C., Wang J., Dong X., Guan R., Cao D., Chen Q. (2019). Non-conjugated organosilicone fluorescent nanoparticles for latent fingerprint detection. J. Lumin..

[117] Zhang L., Liao D., Fan X., Luo B., Jiang L., Qin Y., Liao L., Wang Y., Feng L., Li Z., Qin A. (2025). Dual Photoluminescence Emission Chiral Carbon Quantum Dots for Ratiometric and Visual Fluorescent Ag+ Sensing. ACS Appl. Nano Mater..

[118] Aslam M., Ali S., Hamdy K., Gautam R.K.S., Danishuddin, Ahmad K., Ahmad K. (2025). Progress in Electrode Modifiers for Nitrite Electrochemical Sensing Applications. Biosensors.

[119] Thikra S.D., Yousif Dafhalla A.K., Tayfour O.E., Mubarakali A., Alqahtani A.S., Tayfour Ahmed A.E., Elobaid M.E., Adam T., Gopinath S.C.B. (2024). Advances in nano sensors for monitoring and optimal performance enhancement in photovoltaic cells. iScience.

[120] Tovar-Lopez F.J. (2023). Recent Progress in Micro- and Nanotechnology-Enabled Sensors for Biomedical and Environmental Challenges. Sensors.

[121] Ali E., Ashraf H., Shah S.M.A., Abdullah M. (2025). Enabling Smart and Sustainable Solutions : Applications of Wireless Sensor Networks in the Era of IoT. Int. J. Computational Thinking Data Sci..

[122] Shi Q., Yang Y., Sun Z., Lee C. (2022). Progress of Advanced Devices and Internet of Things Systems as Enabling Technologies for Smart Homes and Health Care. ACS Mater. Au.

[123] Pramanik P.K.D., Solanki A., Debnath A., Nayyar A., El-Sappagh S., Kwak K.S. (2020). Advancing Modern Healthcare with Nanotechnology, Nanobiosensors, and Internet of Nano Things: Taxonomies, Applications, Architecture, and Challenges. IEEE Access.

[124] Nayyar A., Puri V., Le D. (2017). Internet of Nano Things (IoNT): Next Evolutionary Step in Nanotechnology. Nanosci. Nanotechnol..

[125] Zhang Y., Gao S., Li H., Yang T., Zheng K., Guo Z.M., Shi J., Huang X., Zou X., Picchetti P., Biedermann F. (2025). Design Principles of Nanosensors for Multiplex Detection of Contaminants in Food. Small.

[126] Leong Y.X., Tan E.X., Leong S.X., Lin Koh C.S., Thanh Nguyen L.B., Ting Chen J.R., Xia K., Ling X.Y. (2022). Where Nanosensors Meet Machine Learning: Prospects and Challenges in Detecting Disease X. ACS Nano.

[127] Tripathy A., Patne A.Y., Mohapatra S., Mohapatra S.S. (2024). Convergence of Nanotechnology and Machine Learning: The State of the Art, Challenges, and Perspectives. Int. J. Mol. Sci..

[128] Arora S., Nimma D., Kalidindi N., Mary Rexcy Asha S., Eluri N.R., Florence M.M.V. (2024). Integrating Machine Learning With Nanotechnology For Enhanced Cancer Detection And Treatment. South East. Eur. J. Public Health.

[129] Alagumalai A., Shou W., Mahian O., Aghbashlo M., Tabatabaei M., Wongwises S., Liu Y., Zhan J., Torralba A., Chen J. (2022). Self-powered sensing systems with learning capability. Joule.

[130] GÜLEÇ Ö. (2023). Machine Learning Supported Nano-Router Localization in WNSNs. Sakarya University Journal of Science.

[131] Goumas G., Vlachothanasi E.N., Fradelos E.C., Mouliou D.S. (2025). Biosensors, Artificial Intelligence Biosensors, False Results and Novel Future Perspectives. Diagnostics.

[132] Brown K.A., Brittman S., Maccaferri N., Jariwala D., Celano U. (2020). Machine Learning in Nanoscience: Big Data at Small Scales. Nano Lett..

[133] Ji S., Lin C. (2023). Human Motion Pattern Recognition Based on Nano-sensor and Deep Learning. Inf. Technol. Control.

[134] Al Jauhar, Solimun H.S., Fitriani R. (2025). Application of Dbscan for Clustering Society Based on Waste Management Behavior. Barekeng.

[135] Du K.L., Zhang R., Jiang B., Zeng J., Lu J. (2025). Understanding Machine Learning Principles: Learning, Inference, Generalization, and Computational Learning Theory. Mathematics.

[136] J. Kim and J. Kang, (2024) A Theory of Machine Learning, no., pp. 1–24.

[137] Aarif K. O. M., Alam A., Hotak Y. (2025). Smart Sensor Technologies Shaping the Future of Precision Agriculture: Recent Advances and Future Outlooks. J. Sens..

[138] Wan C.H., Hwang M.C. (2018). Value-based deep reinforcement learning for adaptive isolated intersection signal control. IET Intell. Transp. Syst..

[139] Fazzini P., Wheeler I., Petracchini F. (2021). Traffic Signal Control with Communicative Deep Reinforcement Learning Agents: A Case Study.

[140] Raju S.K., Varadarajan G.K., Alharbi A.H., Kannan S., Khafaga D.S., Sundaramoorthy R.A., Eid M.M., Towfek S.K. (2024). Estimating best nanomaterial for energy harvesting through reinforcement learning DQN coupled with fuzzy PROMETHEE under road-based conditions. Sci. Rep..

[141] Sun Y., Yue L., He T., Chen W., Sun Z. (2025). A Bidirectional Material Di ff usion Algorithm Based on Fusion Hypergraph Random Walks for Video Recommendation. Mathematics.

[142] Carone S., Pappalettera G., Casavola C., De Carolis S., Soria L. (2023). A Support Vector Machine-Based Approach for Bolt Loosening Monitoring in Industrial Customized Vehicles. Sensors.

[143] Rajpal D., Mitrotta F.M.A., Socci C.A., Sodja J., Kassapoglou C., De Breuker R. (2021). Design and testing of aeroelastically tailored composite wing under fatigue and gust loading including effect of fatigue on eroelastic performance. Compos. Struct..

[144] Alqudah A.M., Moussavi Z. (2025). Bridging Signal Intelligence and Clinical Insight: A Comprehensive Review of Feature Engineering, Model Interpretability, and Machine Learning in Biomedical Signal Analysis. Appl. Sci..

[145] Huang L., Duan Q., Liu Y., Wu Y., Li Z., Guo Z., Liu M., Lu X., Wang P., Liu F. (2025). Artificial intelligence: A key fulcrum for addressing complex environmental health issues. Environ. Int..

[146] Pillai V.V., Ramasubramanian B., Sequerth O., Pilla S., Wang T., Mohanty A.K., Govindaraj P., Alhassan S.M., Salim N., Kingshott P. (2025). Nanomaterial advanced smart coatings: Emerging trends shaping the future. Appl. Mater. Today.

[147] Khouas A.R., Bouadjenek M.R., Hacid H., Aryal S. (2024). Training Machine Learning models at the Edge: A Survey. J. Latex Class Files.

[148] Luo D., Wang K., Wang D., Sharma A., Li W., Choi I.H. (2025). Arti fi cial intelligence in the design , optimization , and performance prediction of concrete materials : a comprehensive review. NPJ Mater. Sustain..

[149] Cemiloglu A., Zhu L., Arslan S., Xu J., Yuan X., Azarafza M., Derakhshani R. (2023). Support Vector Machine (SVM) Application for Uniaxial Compression Strength (UCS) Prediction: A Case Study for Maragheh Limestone. Appl. Sci..

[150] Guido R., Ferrisi S., Lofaro D., Conforti D. (2024). An Overview on the Advancements of Support Vector Machine Models in Healthcare Applications: A Review. Information.

[151] Thapa A., Aryal N., Reba M.L. (2025). Machine learning models for water quality: Predicting pollutant loads and assessing conservation practice's effectiveness in agricultural fields. Ecol. Inform..

[152] Alimisis V., Gennis G., Gourdouparis M., Dimas C., Sotiriadis P.P. (2023). A Low-Power Analog Integrated Implementation of the Support Vector Machine Algorithm with On-Chip Learning Tested on a Bearing Fault Application. Sensors.

[153] Kim W., Joe H., Kim H.-S., Yoon D. (2024). Interpretable Support Vector Machine and Its Application to Rehabilitation Assessment. Electronics.

[154] Reddi V., Plancher B., Kennedy S., Moroney L., Warden P., Suzuki L., Agarwal A., Banbury C., Banzi M., Bennett M. (2022). Widening Access to Applied Machine Learning with TinyML. Harv. Data Sci. Rev..

[155] Shah V., Yadav P. (2025). Proc. 2025 3rd Int. Conf. Inventive Computing and Informatics (ICICI).

[156] Su Y., Yin D., Zhao X., Hu T., Liu L. (2025). Exploration of Advanced Applications of Triboelectric Nanogenerator-Based Self-Powered Sensors in the Era of Artificial Intelligence. Sensors.

[157] Bhaiyya M., Panigrahi D., Rewatkar P., Haick H. (2024). Role of Machine Learning Assisted Biosensors in Point-of-Care-Testing For Clinical Decisions. ACS Sens..

[158] Warnett S.J., Zdun U. (2022). Proc. 2022 IEEE 19th International Conference on Software Architecture (ICSA).

[159] Kuznetsova V., Coogan Á., Botov D., Gromova Y., Ushakova E.V., Gun’ko Y.K. (2024). Expanding the Horizons of Machine Learning in Nanomaterials to Chiral Nanostructures. Adv. Mater..

[160] Srivastava S., Wang W., Zhou W., Jin M., Vikesland P.J. (2024). Machine Learning-Assisted Surface-Enhanced Raman Spectroscopy Detection for Environmental Applications: A review. Environ. Sci. Technol..

[161] Akyildiz I.F., Kak A., Nie S. (2020). 6G and Beyond: The Future of Wireless Communications Systems. IEEE Access.

[162] Kant K., Beeram R., Cao Y., Dos Santos P.S.S., González-Cabaleiro L., García-Lojo D., Guo H., Joung Y., Kothadiya S., Lafuente M. (2024). Plasmonic nanoparticle sensors: current progress, challenges, and future prospects. Nanoscale Horiz..

[163] Wang B., Li Y., Zhou M., Han Y., Zhang M., Gao Z., Liu Z., Chen P., Du W., Zhang X. (2023). Smartphone-based platforms implementing microfluidic detection with image-based artificial intelligence. Nat. Commun..

[164] Chen J., Yi C., Okegbile S.D., Cai J., Shen X. (2024). Networking Architecture and Key Supporting Technologies for Human Digital Twin in Personalized Healthcare: A Comprehensive Survey. IEEE Commun. Surv. Tutorials.

[165] Mahmmod B.M., Naser M.A., Al-Sudani A.H.S., Alsabah M., Mohammed H.J., Alaskar H., Almarshad F., Hussain A., Abdulhussain S.H. (2024). Patient Monitoring System Based on Internet of Things: A Review and Related Challenges With Open Research Issues. IEEE Access.

[166] Esteban S., Girón-Sierra J.M., Polo Ó.R., Angulo M. (2016). Signal conditioning for the kalman filter: Application to satellite attitude estimation with magnetometer and sun sensors. Sensors.

[167] Foussier J., Teichmann D., Jia J., Misgeld B., Leonhardt S. (2014). An adaptive Kalman filter approach for cardiorespiratory signal extraction and fusion of non-contacting sensors. BMC Med. Inform. Decis. Mak..

[168] Rashidi H.H., Pantanowitz J., Hanna M.G., Tafti A.P., Sanghani P., Buchinsky A., Fennell B., Deebajah M., Wheeler S., Pearce T. (2025). Introduction to Artificial Intelligence (AI) and Machine Learning (ML) in Pathology & Medicine: Generative & Non-Generative AI Basics. Mod. Pathol..

[169] Julian D.R., Bahramy A., Neal M., Pearce T.M., Kofler J. (2025). Current Advancements in Digital Neuropathology and Machine Learning for the Study of Neurodegenerative Diseases. Am. J. Pathol..

[170] Tian C., Gao Y., Rui C., Qin S., Shi L., Rui Y. (2024). Artificial intelligence in orthopaedic trauma. EngMedicine.

[171] Chien A., Lall A., Patel M., Cusumano L., McWilliams J. (2024). Artificial neural networks analysis predicts long-term fistula function in hemodialysis patients following percutaneous transluminal angioplasty. EngMedicine.

[172] Zhu H.D., Liu R., Jia Z.Z., Xia D.D., Zhong B.Y., Fan W.Z., Lu J., Zhao M., Teng G.J. (2024). EngMedicine Transarterial chemoembolization for hepatocellular carcinoma : Treatment algorithm proposed by Chinese College of Interventionalists ( CCI ) ☆ of Chinese College of Interventionalists. EngMedicine.

[173] Lee D., Brown M., Hammond J., Zakowski M. (2025). Readability, quality and accuracy of generative artificial intelligence chatbots for commonly asked questions about labor epidurals: a comparison of ChatGPT and Bard. Int. J. Obstet. Anesth..

[174] Ficili I., Giacobbe M., Tricomi G., Puliafito A. (2025). From Sensors to Data Intelligence: Leveraging IoT, Cloud, and Edge Computing with AI. Sensors.

[175] Giorgetti G., Pau D.P. (2025). Transitioning from TinyML to Edge GenAI: A Review. Big Data Cogn. Comput..

[176] Jouini O., Sethom K., Namoun A., Aljohani N., Alanazi M.H., Alanazi M.N. (2024). A Survey of Machine Learning in Edge Computing: Techniques, Frameworks, Applications, Issues, and Research Directions. Technologies.

[177] Olawade D.B., Ige A.O., Olaremu A.G., Ijiwade J.O., Adeola A.O. (2024). The synergy of artificial intelligence and nanotechnology towards advancing innovation and sustainability - A mini-review. Nano Trends.

[178] Nzeako G., Oladipo Akinsanya M., Adeboye Popoola O., Chukwurah E.G., Okeke C.D. (2025). The role of AI-Driven predictive analytics in optimizing IT industry supply chains. Int. J. Manag. Entrep. Res..

[179] Zhou S., Sun J., Xu K., Wang G. (2024). AI-Driven Data Processing and Decision Optimization in IoT through Edge Computing and Cloud Architecture. Preprint.

[180] Khosravi M., Zare Z., Mojtabaeian S.M., Izadi R. (2024). Artificial Intelligence and Decision-Making in Healthcare: A Thematic Analysis of a Systematic Review of Reviews. Health Serv. Res. Manag. Epidemiol..

[181] Almanasra S. (2024). Applications of integrating artificial intelligence and big data: A comprehensive analysis. J. Intell. Syst..

[182] Panwar V. (2024). AI-Driven Query Optimization: Revolutionizing Database Performance and Efficiency. Int. J. Comput. Trends Technol..

[183] Natarajan R., Ranjith C.P., Mohideen M.K., Gururaj H.L., Flammini F., Thangarasu N. (2024). Utilizing a machine learning algorithm to choose a significant traffic identification system. Int. J. Inf. Manag. Data Insights.

[184] Yaiprasert C., Hidayanto A.N. (2024). AI-powered ensemble machine learning to optimize cost strategies in logistics business. Int. J. Inf. Manag. Data Insights.

[185] Biswas P., Rashid A., Biswas A., Nasim M.A.A., Chakraborty S., Gupta K.D., George R. (2024). AI-driven approaches for optimizing power consumption: a comprehensive survey. Discov. Artif. Intell..

[186] Maleki Varnosfaderani S., Forouzanfar M. (2024). The Role of AI in Hospitals and Clinics: Transforming Healthcare in the 21st Century. Bioengineering.

[187] Nwabueze M.O., Aliyu A., Adegbo K.J., Ikemefuna D. (2024). Enhancing machine optimization through AI-driven data analysis and gathering : leveraging integrated systems and hybrid technology for industrial efficiency. World J. Adv. Res. Rev..

[188] Chen W., Men Y., Fuster N., Osorio C., Juan A.A. (2024). Artificial Intelligence in Logistics Optimization with Sustainable Criteria: A Review. Sustainability.

[189] Aldoseri A., Al-Khalifa K.N., Hamouda A.M. (2023). Re-Thinking Data Strategy and Integration for Artificial Intelligence: Concepts, Opportunities, and Challenges. Appl. Sci..

[190] Alenezi M., Akour M. (2025). AI-Driven Innovations in Software Engineering: A Review of Current Practices and Future Directions. Appl. Sci..

[191] Al-Surmi A., Bashiri M., Koliousis I. (2022). AI based decision making: combining strategies to improve operational performance. Int. J. Production Res..

[192] Richardson N., Kothapalli S., Onteddu A.R., Kundavaram R.R., Talla R.R. (2023). AI-Driven Optimization Techniques for Evolving Software Architecture in Complex Systems. ABC J. ADV. RES..

[193] Moro-Visconti R., Cruz Rambaud S., López Pascual J. (2023). Artificial intelligence-driven scalability and its impact on the sustainability and valuation of traditional firms. Humanit. Soc. Sci. Commun..

[194] Trigka M., Dritsas E. (2025). Edge and Cloud Computing in Smart Cities. Future Internet.

[195] Gupta K., Mane P., Rajankar O.S., Bhowmik M., Jadhav R., Yadav S., Rawandale S., Chobe S.V. (2023). Harnessing AI for Strategic Decision-Making and Business Performance Optimization. Int. J. Intell. Syst. Appl. Eng..

[196] Wu Z., Mao Z., Shen W. (2021). Integrating Multiple Datasets and Machine Learning Algorithms for Satellite-Based Bathymetry in Seaports. Remote Sens..

[197] Wang X., Liu L., Zhang W., Ma X. (2021). Prediction of plant uptake and translocation of engineered metallic nanoparticles by machine learning. Environ. Sci. Technol..

[198] Azab A., Khasawneh M., Alrabaee S., Choo K.K.R., Sarsour M. (2024). Network traffic classification: Techniques, datasets, and challenges. Digital Communications and Networks.

[199] Xu Y., Khan T.M., Song Y., Meijering E. (2025). Edge deep learning in computer vision and medical diagnostics: a comprehensive survey. Artif. Intell. Rev..

[200] Neri L., Oberdier M.T., van Abeelen K.C.J., Menghini L., Tumarkin E., Tripathi H., Jaipalli S., Orro A., Paolocci N., Gallelli I. (2023). Electrocardiogram Monitoring Wearable Devices and Artificial-Intelligence-Enabled Diagnostic Capabilities: A Review. Sensors.

[201] Zhang C., Li J., Guo P., Li Q., Zhang X., Wang X. (2023). A configurable hardware-efficient ECG classification inference engine based on CNN for mobile healthcare applications. Microelectron. J..

[202] Landauer M., Onder S., Skopik F., Wurzenberger M. (2023). Deep learning for anomaly detection in log data: A survey. Machine Learning with Applications.

[203] Ma J., Wang H. (2024). Anomaly detection in sensor data via encoding time series into images. J. King Saud Univ. Comput. Inf. Sci..

[204] Liu R., Wang F., Tian F., Qian J., Chen X., Cui S., Yi L. (2022). MTMI-DCNN: A PSR-Based Method for Time Series Sensor Data Classification. IEEE Sens. J..

[205] Ahmed D.M., Hassan M.M., Mstafa R.J.,A. (2022). Review on Deep Sequential Models for Forecasting Time Series Data. Applied Computational Intelligence and Soft Computing.

[206] Żyliński M., Nassibi A., Rakhmatulin I., Malik A., Papavassiliou C.M., Mandic D.P. (2024). Deployment of Artificial Intelligence Models on Edge Devices: A Tutorial Brief. IEEE Trans. Circuits Syst. II..

[207] Hemdan M., Abuelhaded K., Shaker A.A.S., Ashour M.M., Abdelaziz M.M., Dahab M.I., Nassar Y.A., Sarguos A.M.M., Zakaria P.S., Fahmy H.A. (2025). Recent advances in nano-enhanced biosensors: Innovations in design, applications in healthcare, environmental monitoring, and food safety, and emerging research challenges. Sens. Biosensing Res..

[208] Zhao Y., Ye H., Yang J., Yao S., Lv M., Chen Z., Ye Y., Hu Q., Lu C., Liu Z. (2025). EngMedicine AutoLDP : An accurate and ef fi cient arti fi cial intelligence-based tool for automatic labeling of digital pathology. EngMedicine.

[209] Lu Y., Luo Q., Jia X., Tam J.P., Yang H., Shen Y., Li X. (2023). Multidisciplinary strategies to enhance therapeutic effects of flavonoids from Epimedii Folium: Integration of herbal medicine, enzyme engineering, and nanotechnology. J. Pharm. Anal..

[210] Adir O., Poley M., Chen G., Froim S., Krinsky N., Shklover J., Shainsky-Roitman J., Lammers T., Schroeder A. (2020). Integrating Artificial Intelligence and Nanotechnology for Precision Cancer Medicine. Adv. Mater..

[211] Tripathy A., Patne A.Y., Mohapatra S., Mohapatra S.S. (2024). Convergence of Nanotechnology and Machine Learning: The State of the Art, Challenges, and Perspectives. Int. J. Mol. Sci..

[212] Adam H., Gopinath S.C.B., Kumarevel T., Ashokkumar T., Adam T., Arshad M.K.M., Mohammed M., Voon C.H., Ramli M.M., Uda M.N.A. (2025). Electro-Sensing Analysis for Parkinson's Disease Biomarker on Dual-Electrode Surface: Complemented by Molecular Docking. Biotechnol. Appl. Biochem..

[213] Taha B.A., Abdulrahm Z.M., Addie A.J., Haider A.J., Alkawaz A.N., Yaqoob I.A.M., Arsad N. (2025). Advancing optical nanosensors with artificial intelligence: A powerful tool to identify disease-specific biomarkers in multi-omics profiling. Talanta.

